# Evaluation of Violacein Metabolic Stability and Metabolite Identification in Human, Mouse, and Rat Liver Microsomes

**DOI:** 10.3390/pharmaceutics17050601

**Published:** 2025-05-02

**Authors:** Debora Bressanim de Aquino Calemi, Alexandre Barcia Godoi, Giulia Minuti, Fausto Carnevale Neto, Gabriel Felipe Hispagnol, Alan Cesar Pilon, Jose Luiz Costa, Stephen Hyslop, Natalicia de Jesus Antunes

**Affiliations:** 1 Departamento de Farmacologia, Faculdade de Ciências Médicas, Universidade Estadual de Campinas (UNICAMP), Campinas 13083-888, SP, Brazil; deboracalemi@gmail.com (D.B.d.A.C.); bdgalexandre@gmail.com (A.B.G.); giu.minuti@gmail.com (G.M.); josejlc@unicamp.br (J.L.C.); hyslop@unicamp.br (S.H.); 2 Centro de Informação e Assistência Toxicológica (CIATox) de Campinas, Universidade Estadual de Campinas (UNICAMP), Campinas 13083-888, SP, Brazil; 3 Northwest Metabolomics Research Center, Department of Anesthesiology and Pain Medicine, University of Washington, 850 Republican Street, Seattle, WA 98109, USA; fcarnevaleneto@gmail.com; 4 Departamento de Bioquímica e Química Orgânica, Instituto de Química, Universidade Estadual Paulista, Araraquara 14800-060, SP, Brazil; gabriel.hispagnol@unesp.br (G.F.H.); alan.pilon@unesp.br (A.C.P.); 5 Faculdade de Ciências Farmacêuticas, Universidade Estadual de Campinas (UNICAMP), Campinas 13083-859, SP, Brazil

**Keywords:** ADME, drug development, liver microsomes, malaria, mass spectrometry, metabolic stability, metabolite profiling and stability, violacein

## Abstract

**Background**: Malaria significantly impacts the health of populations living in poverty and vulnerable conditions. Resistance to current antimalarial drugs remains a major challenge and highlights the urgent need for novel, effective, and safer therapies. Violacein, a purple pigment, has demonstrated potent antiplasmodial activity, making it a promising antimalarial candidate. However, to date, no in vitro metabolism studies of violacein have been published. In this study, the metabolic stability of violacein was evaluated using human (HLMs), mouse (MLMs), and rat (RLMs) liver microsomes and the metabolites generated by HLMs and RLMs were assessed. **Methods**: Liquid chromatography quadrupole mass spectrometry (LC-MS/MS) was used to investigate the metabolic stability of violacein, while liquid chromatography quadrupole time-of-flight mass spectrometry (LC-QTOF-MS) was used to identify the metabolites. In silico analyses were used to support in vitro metabolite identification by providing insights into potential metabolic pathways and predicting metabolite structures, thereby enhancing the accuracy and efficiency of the identification process. **Results**: The half-life (t_1/2_) for violacein in RLMs, MLMs, and HLMs was 36, 81, and 216 min, respectively. The in vitro intrinsic clearance (CL_int, in vitro_) values were 38.4, 17.0, and 6.4 µL/min/mg for RLMs, MLMs, and HLMs, respectively, while the in vivo intrinsic clearance (CL_int, in vivo_) was 93.7, 67.0, and 6.6 mL/min/kg, respectively. A slow elimination profile was observed in HLMs followed by MLMs, with rapid elimination in RLMs, indicating greater stability of violacein in HLMs and MLMs when compared with RLMs. Four violacein metabolites were identified in HLMs and RLMs, two of which were formed by phase I metabolism, one by phase II metabolism, and one by phase I + II metabolism. **Conclusions**: This study provides the first published analysis of the metabolic stability of violacein.

## 1. Introduction

Malaria is an infectious disease caused by protozoan parasites of the genus *Plasmodium*, with *Plasmodium falciparum* generally considered the most important species in terms of deaths. Mammalian infection starts with the injection of sporozoites during the blood feed of an infected female *Anopheles* mosquito. Malaria continues to be one of the most significant public health challenges worldwide, particularly in tropical and subtropical regions [1,2]. This disease affects primarily people living in vulnerable circumstances, often without access to adequate housing, proper environmental management, essential medicines, insecticides, and mosquito nets. According to the latest World Malaria Report from the World Health Organization (WHO), over 263 million cases of malaria were reported in 2023, representing an increase of 11 million cases compared to 2022, although the number of deaths has remained relatively unchanged (600,000 in 2022 and 597,000 in 2023) [3,4]. The greatest challenge in combating and controlling malaria is the growing resistance of *Plasmodium* ssp. to established treatments, particularly since resistance not only reduces the effectiveness of treatments but also prolongs the infection and increases the risk of more severe complications [5,6]. In addition to resistance caused by the parasite’s ability to survive and/or multiply as a result of spontaneous mutations in specific genes, drug resistance also depends on factors such as transmission dynamics in different geographic areas, immunity, parasite load, and pharmacokinetic (PK) and pharmacodynamic (PD) properties of the antimalarials. This situation highlights the urgent need to develop new, more effective, and safer treatments of malaria [7,8].

Violacein (3-[1,2-dihydro-5-(5-hydroxy-1H-indol-3-yl)-2-oxo-3H-pyrrole-3-ylidene-1,3-dihydro-2H-indol-2-one) is a purple pigment biosynthesized by various Gram-negative bacteria, particularly *Chromobacterium violaceum*. Violacein has a variety of biological activities, including antibacterial, antifungal, antineoplastic, antiviral, antioxidant, and antiprotozoal activities, particularly antiplasmodial activity [9,10,11,12,13,14,15]. Potent antibacterial activity has been demonstrated for violacein, particularly against Gram-positive bacteria, with its efficacy against *Staphylococcus aureus* being well established (minimum inhibitory concentrations, MICs: 6.25 to 30 μM). The bactericidal mechanism of violacein involves direct interaction with bacterial membrane components, leading to membrane disruption, loss of cellular homeostasis, and, ultimately, cell lysis. This membrane-targeting activity is attributed to the lipophilic nature of violacein that facilitates insertion into the lipid bilayer and compromises membrane integrity [16].

Violacein exerts strong antifungal activity against a range of human, plant, and yeast pathogens, with MICs of 2 μg/mL for *Trichophyton rubrum*, 8 μg/mL for *Candida albicans*, 16 μg/mL for *Candida tropicalis*, and 32 μg/mL for *Cryptococcus gastricus*. In some cases, violacein outperformed conventional antifungal agents such as bavistin and amphotericin B. Although the precise mechanism remains to be fully elucidated, the antifungal activity is likely associated with membrane disruption, leading to the loss of intracellular contents and cell death [17]. The antifungal potential was also evaluated using computational modeling, specifically targeting *Aspergillus fumigatus*. Molecular docking analysis and molecular dynamics simulations demonstrated a robust and stable interaction between violacein and nucleoside diphosphate kinase (Ndk), an enzyme essential for nucleotide synthesis in this fungus [18].

Violacein exhibited potent cytotoxic activity against colorectal cancer cells, with Caco-2 cells being particularly sensitive. Treatment induced apoptosis through increased production of reactive oxygen species (ROSs), oxidative stress, mitochondrial membrane disruption, and cytochrome c release. The IC_50_ for Caco-2 cells was approximately 2 μM, whereas HT29 cells showing marked resistance (IC_50_ > 10 μM). These findings underscore the potential of violacein as a promising candidate for antitumor therapy, especially in ROS-sensitive cancer phenotypes [19].

In a murine model of experimental autoimmune encephalomyelitis (EAE), violacein significantly attenuated disease severity and reduced the maximum clinical score from 4 to 2 after three days of treatment with intraperitoneal doses of 3.5 mg/kg. This improvement was associated with a marked decrease in pro-inflammatory cytokines (IL-17, IFN-γ, and TNF-α) in the central nervous system, alongside an increase in regulatory T cell populations (CD4^+^CD25^+^FOXP3^+^). Furthermore, violacein treatment reduced the expression of pro-inflammatory mediators such as TNF-α and IL-6 in acute and chronic inflammation models. Although not directly assessed, these effects may suggest an impact on macrophage function, potentially contributing to a more anti-inflammatory environment and promoting immune homeostasis [11]. Violacein exhibits immunomodulatory properties that reduce glial inflammation and promote a neuroprotective environment. Treatment with violacein significantly decreased pro-inflammatory cytokines such as TNF-α, IL-1β, and IL-6 in the spinal cord of rats with amyotrophic lateral sclerosis (ALS). Violacein also inhibited the activity of matrix metalloproteinases MMP-2 and MMP-9, enzymes implicated in ALS progression and motor neuron degeneration. A weekly intraperitoneal dose of 300 nmol/kg resulted in modest improvements in survival and the preservation of neuromuscular junction integrity. These neuroprotective effects are likely mediated by violacein’s ability to modulate glial reactivity, attenuate oxidative stress, and preserve mitochondrial function [20]. These studies indicate that violacein has remarkable potential as a valuable bioactive compound for future innovations and therapeutic developments [9].

In malaria research, violacein has received increasing attention because of its promising antimalarial properties, making it a compelling candidate for novel treatment strategies. Violacein is active against *P. falciparum*, with an IC_50_ of ~0.4 µM against the 3D7 strain (chloroquine-sensitive) of the parasite and 0.5 µM against the W2 strain (chloroquine-resistant), indicating that the compound has similar efficacy against both chloroquine-sensitive and chloroquine-resistant strains [14,15]. This finding suggests that chloroquine resistance is not part of the action of violacein [14]. Violacein affects protein homeostasis of *P. falciparum*. Mechanistically, violacein binds to the *P. falciparum* chaperones PfHsp90 and PfHsp70-1, compromising the adenosine triphosphatase (ATPase) and chaperone activity of the latter. Furthermore, violacein-treated parasites exhibited increased protein unfolding and proteasomal degradation. The uncoupling of the parasite stress response reflects the multistage growth inhibitory effect promoted by violacein [15]. C57BL/6 mice treated for three consecutive days with violacein (3.75 mg/kg/day) presented a 82% reduction in parasitemia [21].

Together, these findings indicate that violacein is a potentially useful and versatile compound for the development of new antimalarial drugs to deal with the growing challenge of drug resistance, especially the emergence of partial resistance to artemisinin in artemisinin-based combination therapies (ACTs), already identified in regions of Asia and Africa where malaria is endemic [22,23].

As new compounds are selected in the discovery process, it is essential to evaluate their pharmacokinetic properties, including their profiles of absorption, distribution, metabolism, and excretion (ADME) [24,25]. In vitro drug metabolism studies are important to determine the metabolic stability, to identify metabolites, and to elucidate metabolic pathways of a compound. Metabolic stability studies, which are performed to estimate the susceptibility of a compound to metabolism, generate results that are used to predict in vivo pharmacokinetic parameters such as bioavailability, half-life (t_1/2_), and intrinsic clearance (CL_int, in vivo_). If a compound is rapidly metabolized, its bioavailability will probably be low, leading to a decrease in the therapeutic efficacy [26,27].

The identification of metabolites provides insights into the metabolic degradation of a drug, helps identify the sources of metabolic instability, and enhances the understanding of metabolic pathways [28]. Such preliminary in vitro experiments are useful for selecting the appropriate animal models for subsequent studies in vivo and for identifying suitable surrogate species for humans. This approach helps to ensure both cost and time efficiency of future experiments [29,30,31,32,33]. These experiments also provide essential insights for in vivo mass balance studies, enabling the evaluation of safety, efficacy, and personalized treatments while optimizing dosing regimens, minimizing adverse effects, and enhancing the development of new formulations [34,35].

Drug metabolism involves the conversion of a lipophilic compound to more hydrophilic metabolites to facilitate its elimination from the body. This process is divided into phase I and II reactions. Phase I metabolism involves oxidation, reduction, and hydrolysis and these reactions generally result in minimal changes to the molecular weight or aqueous solubility of the compound. Cytochrome P450 (CYP450) enzymes are the main enzymes responsible for phase I metabolism. Key CYP450 isoforms involved in drug metabolism include CYP1A2, CYP2C9, CYP2C19, CYP2D6, CYP2A6, CYP2E1, and CYP3A4. The UDP-glucuronosyltransferases (UGTs), sulfotransferases (SULTs), and methyltransferases (MTs) are the main enzymes responsible for phase II metabolism, which involves reactions such as glucuronidation, sulfation, methylation, and others. These processes typically involve the conjugation or unmasking of functional groups, often polar groups such as –OH, –SH, or –NH_2_, of the parent molecule or of the phase I metabolite. This significantly increases the molecule’s polarity, enhancing its solubility and facilitating its excretion from the body [36,37,38].

Among the available in vitro models, especially in metabolic stability studies, liver microsomes are a good model because of their ease of use and enzyme profile that includes key CYP450 family enzymes and UGTs. Consequently, liver microsomes are particularly suitable for detecting phase I and phase II metabolites. Additionally, microsomes thawed and kept on ice for <2 h can be refrozen at −80 °C without loss of enzymatic activity, thereby ensuring stability and reliability in in vitro metabolism studies. Although hepatocytes, plasma, and the S9 fraction are also commonly used, microsomes remain an efficient option because of their enzymatic content and high stability [38,39]. Mass spectrometry (MS) is a powerful and indispensable tool for studying in vitro metabolism, especially when combined with liquid chromatography (LC-MS/MS) [40]. This combination allows precise identification of metabolites with high accuracy and detailed structural information through fragmentation profiles. Quadrupole time-of-flight mass spectrometry (QTOF-MS) is particularly useful for analyzing biological samples, such as liver microsomes, hepatocytes, plasma, and urine, and can provide a complete picture of compound metabolism, with the identification of phase I and II metabolites. QTOF-MS can also provide reliable data on compound degradation and metabolite formation, thereby aiding in the prediction of bioavailability and pharmacokinetic behavior [40,41,42,43,44]. In addition, QTOF-MS can be used to identify the main chemical constituents present in plant extracts, as demonstrated in the study by Lou et al. [45], who used UPLC–TOF/MS to characterize the major components, including quercetin and kaempferol glycosides, in an extract of *Botrychium ternatum*.

The integration of in silico approaches in drug development enhances the prediction of metabolic pathways and facilitates the selection of compounds with more favorable pharmacokinetic and toxicological profiles, thereby significantly reducing the time and costs involved [24,46,47,48,49,50].

This study investigated, for the first time, the interspecies variation in the in vitro metabolic stability of violacein using human (HLMs), mouse (MLMs), and rat (RLMs) liver microsomes. Violacein stability was analyzed using a liquid chromatography quadrupole mass spectrometry (LC-MS/MS) method. The in vitro intrinsic clearance (CL_int, in vitro_) obtained was subsequently extrapolated to the in vivo setting using allometric scaling. The violacein metabolites produced by phase I metabolism involving CYP450 enzymes and phase II involving UGT enzymes in HLMs and RLMs were identified by liquid chromatography quadrupole time-of-flight mass spectrometry (LC-QTOF-MS). In addition, in silico platforms were used for the preliminary evaluation and predictive analysis of physicochemical properties and metabolic pathways of violacein, as part of the identification of metabolites generated in vitro.

## 2. Materials and Methods

This study protocol was adapted from the protocols described by Godoi et al. [43] and Antunes et al. [51], while ensuring that they complied with the best practice guidelines for drug metabolism studies [39] and the ICH M12 guideline on Drug Interaction Studies (2024) [52].

### 2.1. Chemicals and Reagents

The reference standard violacein from the bacterium *Janthinobacterium lividum* (98% purity, with a minimum violacein content of 85% and a maximum deoxyviolacein content of 15%), omeprazole (used as an internal standard, IS), methanol, acetonitrile, ammonium formate, glucose-6-phosphate, β-nicotinamide adenine dinucleotide phosphate hydrate (NADP^+^), uridine 5-diphosphoglucuronic acid trisodium salt (UDPGA), adenosine 3-phosphate 5-phosphosulfate triethylammonium salt (PAPS), S-(5-adenosyl)-L-methionine p-toluenesulfonate salt (SAM), magnesium chloride hexahydrate, and sodium citrate tribasic dihydrate were purchased from Sigma-Aldrich (St. Louis, MO, USA). Dapaconazole (free base), used as a positive control for the incubations, was supplied by Biolab Farmacêutica (São Paulo, SP, Brazil). Corning^®^ Gentest™ 0.5 M phosphate buffer, pH 7.4 and glucose-6-phosphate dehydrogenase were purchased from Corning (Woburn, MA, USA) and formic acid was acquired from Scharlab (Sentmenat, Barcelona, Spain). Ultrapure water was obtained using a Milli-Q RG system (Millipore, Burlington, MA, USA). LC/MS grade water and LC/MS grade methanol were purchased from Merck (Darmstadt, Germany).

#### Preparation of Stock Solutions and Microsomes

A stock solution of violacein was prepared by dissolving 1 mg of reference standard in 3 mL of methanol, yielding a concentration of 843 µM (290 µg/mL). A working solution of 100 µM violacein was prepared by appropriate dilution of stock solution in 100 mM phosphate buffer, pH 7.4, for the metabolic stability study. Dapaconazole (2.4 mM, 1 mg/mL) was prepared in methanol and omeprazole (10 µM, 1.74 µg/mL) was prepared in acetonitrile.

The HLMs containing 20 mg of microsomal protein/mL and 270 pmol CYP450/mg of protein were obtained from Sigma-Aldrich and stored at −80 °C until use. The RLMs and MLMs were isolated by differential ultracentrifugation, as described by Godoi et al. [43], and the microsomal protein concentration was determined using the Bradford method [53]. Livers were obtained from healthy male mice and rats that had been euthanized for other experiments in the Department of Pharmacology of the State University of Campinas (UNICAMP). All experimental protocols were performed according to the Ethical Principles for Animal Research of the Brazilian Society for Laboratory Animal Science (SBCAL) and were approved by the institutional Committee for Ethics in Animal Use at UNICAMP (CEUA-UNICAMP) as part of a prior project, under protocol number 5987-1/2022 for rats and 5865-1/2022 for mice.

After removal, a pool consisting of livers from five naïve male rats and five C57BL male mice was immediately placed in a 50 mM Tris-HCl buffer, pH 7.4, containing 150 mM KCl. The livers were diced with scissors and washed three times with buffer, followed by homogenization with 20 mL of buffer in a Potter-type homogenizer (three cycles, with each cycle comprising three grindings of 1 min each at 1000 rpm). The homogenate was centrifuged at 10,000× *g* for 15 min at 4 °C. The resulting supernatant was then ultracentrifuged at 100,000× *g* for 60 min at 4 °C to obtain the microsomal pellet, which was resuspended in 50 mM HEPES-HCl buffer, pH 7.4, containing 20% glycerol and 1 mM EDTA.

### 2.2. Microsomal Incubation

#### 2.2.1. Microsomal Cofactors

Phase I cofactors: The NADPH-regenerating system used in this study consisted of 1.1 mM NADP^+^, 10 mM glucose-6-phosphate, 1 U/mL glucose-6-phosphate dehydrogenase, 5 mM sodium citrate, and 66 mM magnesium chloride, all dissolved in 100 mM phosphate buffer (pH 7.4). This system was selected for phase I reactions because of its essential role in supporting the catalytic activity of cytochrome P450 enzymes, which require a continuous supply of electrons to perform redox reactions. NADP^+^, the oxidized form of NADPH, serves as the key electron donor cofactor, ensuring sustained enzymatic activity throughout the experiment [38,39].

Phase II cofactors: Although the presence of SULTs and methyltransferases in microsomes is unlikely, the possibility of trace amounts due to cytosolic contamination cannot be completely ruled out. To ensure a comprehensive approach and maximize the detection of all possible conjugates, the following additional cofactors were included: PAPS, used by SULTs and essential for sulfation reactions that add sulfate groups (SO_3_^−^), SAM, the methyl donor used by methyltransferases in methylation reactions, and UDPGA, a key cofactor for UGTs, which catalyze glucuronidation by adding glucuronic acid to substrates. While UGTs are known to be present in liver microsomes, the inclusion of UDPGA ensures sufficient cofactor availability to support these phase II conjugation reactions [38,39].

#### 2.2.2. In Vitro Metabolic Stability of Violacein in HLMs, MLMs, and RLMs

The metabolic stability of volacein was assessed as described by Godoi et al. [43] and Antunes et al. [51]. Aliquots (4 µL) of 100 µM violacein working solution and 96 µL of the NADPH-regenerating system, containing 1.1 mM NADP^+^, 10 mM glucose-6-phosphate, 1 U/mL glucose-6-phosphate dehydrogenase, 5 mM sodium citrate, and 66 mM magnesium chloride in 100 mM phosphate buffer, pH 7.4, were transferred to propylene tubes and pre-incubated for 5 min in an MTC 100 thermo shaker incubator (Miulab, Zhejiang, China) at 300 rpm and 37 °C. Subsequently, the metabolic reactions were initiated by adding 100 μL of microsomal proteins from HLMs, RLMs, and MLMs at a concentration of 2 mg/mL to the pre-incubated shaking tubes, resulting in final concentrations of 2 μM for violacein and 1 mg/mL for microsomal proteins. In this experiment, seven incubation times were planned for each species (0, 3, 5, 15, 30, 60, and 120 min), in triplicate. At each time interval, the reactions were stopped by adding 400 μL of ice-cold acetonitrile spiked with 10 µM omeprazole (IS) to each tube. Finally, the samples were vortexed for 5 min in a BenchMixer^TM^ XL (Benchmark, NJ, USA) and centrifuged at 12,000× *g* for 15 min at 4 °C (Hettich^®^ Universal 320 R, Tuttlingen, Germany). The supernatants were transferred to a 96-well plate and 8 μL was injected into the LC-MS/MS system (see Section 2.3).

All incubations were performed simultaneously with positive and negative controls. The positive controls were prepared by incubating dapaconazole at the same conditions as violacein (final concentrations: 2 μM) and its metabolic stability was compared with previous studies [39,43,44,51,54]. Similarly, the negative controls were prepared by incubating violacein in buffer solution, in the absence of microsome and cofactor solutions.

#### 2.2.3. Identification of Violacein Metabolites in HLMs and RLMs

The metabolism of violacein was investigated using HLMs and RLMs, by incubating the samples for 120 min under four conditions (*n* = 1): zero sample, phase I-only incubation, phase I and II incubation, and phase II-only incubation, as described by Godoi et al. [43].

For all samples, 25 µL of the 843 µM violacein stock solution was added to propylene tubes and evaporated to dryness under a nitrogen stream. The dried residue was then reconstituted with 2 µL of methanol, ensuring that the final organic solvent concentration remained below 1% to minimize any potential impact on microsomal activity, as described by Jia et al. [39].

To assess phase I metabolism, 148 μL of an NADPH-regenerating system containing 1.1 mM NADP+, 10 mM glucose-6-phosphate, 1 U/mL glucose-6-phosphate dehydrogenase, 5 mM sodium citrate, and 66 mM magnesium chloride in 100 mM phosphate buffer (pH 7.4) were added to the tubes. The solutions were pre-incubated for 5 min at 37 °C and 300 rpm in an MTC 100 thermo shaker incubator, and metabolic reactions were initiated by adding 50 μL of HLMs or RLMs (20 mg/mL), to yield final concentrations of 105 µM violacein and 5 mg of microsomal protein/mL. After 120 min of incubation, the reactions were stopped with 200 μL of ice-cold acetonitrile, vortexed for 5 min (BenchMixer™ XL), and centrifuged at 12,000× *g* for 15 min at 4 °C (Hettich^®^ Universal 320 R centrifuge). The supernatants were transferred to vials, and 10 μL was injected into an LC-QTOF system for analysis (see Section 2.4).

To assess phase II-only metabolism, the NADPH-regenerating system was replaced with 148 µL of a phase II cofactor solution containing 13.70 mM UDPGA, 0.49 mM PAPS, and 3.79 mM SAM in 100 mM phosphate buffer (pH 7.4). The NADPH-regenerating system was omitted to ensure that only direct conjugation reactions occurred without prior phase I modifications. For the combined metabolism of phases I and II, the NADPH regeneration system and phase II cofactors were added, to allow sequential metabolism first by phase I enzymes followed by phase II conjugation reactions. The incubation conditions, reaction termination, and sample processing steps were identical to those described for phase I metabolism, with final concentrations of 105 µM violacein and 5 mg of microsomal protein/mL.

To compare with incubated samples from HLMs and RLMs, zero samples were prepared under identical conditions but reactions were immediately stopped to prevent metabolism.

### 2.3. Analysis of Violacein for Metabolic Stability by LC-MS/MS

Violacein was analyzed using LC-MS/MS (LC-QQQ MS, LCMS8045, Shimadzu, Kyoto, Japan). Chromatographic separation was achieved on a Raptor^TM^ Biphenyl column (2.1 × 100 mm, 2.7 μm) at 40 °C. The mobile phases consisted of water (mobile phase A) and methanol (mobile phase B), both containing 2 mM ammonium formate and 0.1% (*v/v*) formic acid. A flow rate of 0.3 mL/min was used with an elution gradient starting at 20% mobile phase B and holding for 0.2 min, followed by a linear increase to 95% mobile phase B over 2 min that was then maintained for 1 min, with a return to the initial condition in 0.2 min. The total chromatographic run time was 5 min.

The mass spectrometer was operated in positive electrospray ionization mode (ESI^+^), with the following parameters: 4.5 kV electrospray voltage, 3 L/min nebulizing gas (N_2_), 15 L/min heating gas (N_2_), 5 L/min drying gas (air), 400 °C interface temperature, 200 °C desolvation line temperature, 400 °C heating block temperature, and 270 kPa collision-induced dissociation (CID) gas pressure.

The analysis was performed in multiple reaction monitoring (MRM) mode, selecting *m*/*z* 344.0 > 301.0 as the quantifying transition for violacein (collision energy, CE = 27 eV) and *m*/*z* 344.0 > 271.0 as the confirmatory transition (CE = 39 eV). Dapaconazole (*m*/*z* 415.0 > 159.0, CE = 32 eV) was monitored as a positive control, omeprazole was monitored as the IS (*m*/*z* 346.0 >198.0, CE = 12 eV), and deoxyviolacein (*m*/*z* 328.0 > 285.0, CE = 25 eV) was monitored because of its presence in the standard. Data acquisition was done using LabSolutions 5.10 and processed with LabSolutions Insight 3.8 (Shimadzu).

### 2.4. Identification of Violacein and Its Metabolites by LC-QTOF

Violacein and its metabolites were identified using LC-QTOF (LCMS9030, Shimadzu, Kyoto, Japan). Chromatographic separation was performed on a Cortecs T3 C18 column (2.1 × 150 mm, 2.7 μm, Waters) at 40 °C. The mobile phases consisted of LC/MS-grade water (mobile phase A) and LC/MS-grade methanol (mobile phase B), both containing 0.1% (*v*/*v*) formic acid. A flow rate of 0.3 mL/min was used with an elution gradient starting at 5% mobile phase B and holding for 1 min, followed by a linear increase to 95% mobile phase B over 18 min that was then maintained for 3 min, with a return to the initial condition in 0.1 min. The total chromatographic run time was 25 min.

The mass spectrometer was operated in both positive and negative electrospray ionization modes (ESI^+^ and ESI^−^), with the following parameters: 4.5 kV (positive mode) and −3.5 kV (negative mode) electrospray voltage, 10 L/min heating gas (air), 10 L/min drying gas (air), 2 L/min nebulizer gas (N_2_), 400 °C interface temperature, and 250 °C desolvation line temperature.

Data were acquired in data-dependent acquisition (DDA) mode by performing a full scan from *m*/*z* 50 to 800 while triggering top-five fragmentation events for precursors exceeding 1,000 counts (*m*/*z* 60 to 800, collision energy ranging from 30 ± 25 eV). The mass spectrometer was calibrated using a sodium iodide (Na-(NaI)_5_) solution as a mass reference standard (*m*/*z* 1971.6144), ensuring a mass error <1 ppm and a minimum resolution of 30,000. Data were acquired using LabSolutions 5.10 software and processed with LabSolutions Insight Explorer 1.0.0.0 software (Shimadzu).

### 2.5. In Silico Prediction of the Physicochemical Properties and Metabolism of Violacein

Meteor Nexus (v.3.1.0, Lhasa Limited, Leeds, UK) knowledge-based expert systems (Knowledge Bases: Meteor KB 2015 1.0.0) provides precise metabolic predictions for a molecule. A demo license for the software was used to perform predictions in this study. For the prediction of metabolites, metabolic processing parameters were configured as follows: processing direction—breadth first; phase constraint—grow from phase II products; metabolic cycles—limited to a maximum depth of three, using the following prediction method options: site of metabolism scoring (with molecular mass variance), with a molecular mass similarity threshold of 70, a scoring filter set at “relative”, and a score threshold of 70. The maximum number of metabolites was set to 1,000, and mammals were the species of choice. Meteor Nexus uses a machine learning-based site of metabolism prediction and combines this with reaction rules to predict and rank the structures of metabolites potentially formed by phase I and/or phase II metabolism [55,56,57,58]. The following reaction types were considered for phase I metabolism: redox reactions—oxidation and reduction; non-redox reactions—hydrolysis, dehydration and hydration, hydrolytic and non-hydrolytic fragmentation or ring opening, ring closure, decarboxylation. In addition, for phase II metabolism, the following reactions were included: glucuronidation reactions—O-glucuronidation, N-glucuronidation, and S-glucuronidation; and glucuronidation conjugation—reactions involving alkyl halides, halogenated alkenes, alkynes, esters, epoxides, episulfites, arene oxides, and oxygen conjugates.

GastroPlus^®^ version 10.1 (Simulations Plus Inc., Lancaster, CA, USA) was used for in silico predictions. The ADMET Predictor^®^ module (version 12.0) was employed to predict physicochemical properties such as lipophilicity, measured by the partition coefficient (log P), molecular weight, solubility, pKa, and metabolism of violacein. To use the ADMET Predictor^®^, a ‘Violacein Database’ was created and an SDF structure file was imported. After loading, the ‘Import Structure Properties’ window appeared, where the ‘Use Predicted’ option was selected to apply ADMET Predictor^®^ data to the uploaded structure. For phase I metabolism reactions, the following enzymes were selected: CYP1A2, CYP2A6, CYP2B6, CYP2C8, CYP2C9, CYP2C19, CYP2D6, CYP2E1, CYP3A4, and aldehyde oxidase (AOX). For phase II metabolism reactions, the following enzymes were selected: UDP-glucuronosyltransferase 1A1 (UGT1A1), UGT1A3, UGT1A4, UGT1A6, UGT1A8, UGT1A9, UGT1A10, UGT2B7, and UGT2B15. The MedChem Designer^®^ module (version 8.0) was used to obtain the chemical structures, formulas, and identifiers (SMILES) of the violacein metabolites.

The violacein fragments were predicted and assigned using *Competitive Fragmentation Modeling for Metabolite Identification* (CFM-ID) [59]. Using the CFM-ID prediction, the *m*/*z* values corresponding to the fragment ions were analyzed in the fragmentation spectra to confirm the potential metabolites formed. In addition, possible chemical reactions were investigated based on the enzymes present in the incubation system. The exact mass calculations were performed using the exact mass calculator of the MS Online Tools of Scientific Instrument Services (https://www.sisweb.com/referenc/tools/exactmass.htm (accessed on 20/01/2025)).

### 2.6. Data Analysis

#### 2.6.1. In Vitro Metabolic Stability of Violacein

In vitro t_1/2_ was calculated using to the following equation [60,61]:

(1) $$t_{1/2}=\frac{ln(2)}{k}$$

where k is the slope of the log-linear regression graph of the percentage of drug remaining as a function of time.

CL_int, in vitro_ was calculated using to the following equation [60,61]:

(2) $${CL}_{int,\;in\;vitro}=\frac{0.693}{t_{1/2}}\times \frac{V_{incubation}}{m_{microsomes}}$$

where V_incubation_ indicates the volume of the incubation in μL and m_microsomes_ indicates the mass of microsomal proteins added to the incubation solution in mg.

After determining CL_int, in vitro_, it was possible to calculate CL_int, in vivo_ using allometric scaling, as follows [29,30,31,32]:

(3) $${CL}_{int,\;in\;vivo}={CL}_{int,\;in\;vitro}\times \frac{m_{microsomes}}{g_{liver}}\times \frac{m_{liver}}{{kg}_{per\;body\;weight}}$$

where m_microsomes_ is 61, 45, and 40 mg and represents the mass of microsomal protein per gram of liver (g_liver_) for rats, mice, and humans, respectively. The m_liver_ is 40, 87.5, and 25.7 g/kg and represents the mass of liver per kilogram of body weight (kg_per body weight_) for rats, mice, and humans, respectively [29,30,31,32].

#### 2.6.2. Identification of Violacein and Its Metabolites

The acquired chromatograms and mass spectra were analyzed using Insight Explore software version 1.0.0.0 (Shimadzu, Kyoto, Japan). To accept the fragmentation spectrum of the metabolite, only ions with *m*/*z* values corresponding to compounds with theoretical structures compatible with the major phase I and/or phase II metabolic pathways were considered. For the identification of violacein and its metabolites, only ions for which the mass error was within 5 ppm for precursor ions and 10 ppm for product ions were accepted. To characterize the structures of the metabolites, only spectra containing at least two ions, in addition to the precursor ion, consistent with their expected fragmentation patterns were considered. Spectra that did not meet these criteria were excluded from metabolite characterization.

## 3. Results

### 3.1. In Silico Prediction of the Physicochemical Properties of Violacein by ADMET Predictor^®^

The physicochemical properties of violacein, predicted based on its molecular structure, are summarized in Table 1. The software-calculated molecular weight was 343.344 g/mol, while the predicted lipophilicity (S + log P) was 3.226. The predicted water solubility (S + Sw) was 0.005 mg/mL, and the predicted effective permeability (S + Peff) in the human jejunum was 1.208 cm^2^/s.

The MedChem Designer^®^ module was used to determine the pKa through a microstate pKa analysis that accounts for the different protonation states of the molecule (Figure S1) [62]. Due to the presence of multiple ionizable groups in the violacein structure, various thermodynamic energy states (microstates) contribute to its overall pKa. During metabolism, structural modifications—such as the addition of hydroxyl groups—can alter the pKa, thereby increasing water solubility and facilitating excretion. In simulated prediction models, microstate pKa analysis enhances the accuracy of molecular ionization assessments. The predicted pKa values for microstates ranged from 11.28 to 10.21 [62].

### 3.2. In Silico Prediction of In Vitro Metabolism of Violacein

The Meteor Nexus generated a total of 38 metabolites. However, the predicted metabolites were ranked based on their similarity scores (threshold). For this evaluation, only metabolites with scores above 70 were considered. Among the high-similarity metabolites, those with the highest intensity and biological relevance were selected. A higher score indicates that the experimental spectrum is closer to the theoretical spectrum of the predicted metabolite, thereby increasing confidence in the identification. Among them, 25 metabolites were selected for analysis. The majority underwent phase II metabolism, with glucuronidation being the most frequent reaction, followed by reduction and hydroxylation. The predicted metabolites and their corresponding reactions are listed in Table S1.

Under the given conditions, ADMET Predictor^®^ successfully identified the formation of 12 phase I metabolites, resulting from either oxidation or reduction reactions. Hydroxylation at different positions of the benzene ring and phenol oxidation were prominent among these reactions. Additionally, the software predicted the potential formation of three phase II metabolites through conjugation with glucuronic acid. Reduction reactions for metabolites M1, M3, M5, M7, M9, M10, M11, and M12 were primarily mediated by CYP1A2, CYP2C9, and/or CYP3A4, with variations for each metabolite. Meanwhile, oxidative metabolism was predicted for M2, M4, M6, and M8, involving CYP1A2, CYP2C9, and/or CYP3A4, with M8 specifically processed by CYP3A4. The product formed was violacein glucuronide, resulting from the conjugation of glucuronic acid to the phenol through the glucuronidation of aromatic alcohols (M1) or to the secondary amide through the glucuronidation of aromatic nitrogen (M2). The predicted metabolites, their corresponding reactions, and the enzymes identified by the software are presented in Table S2 and Figure S2.

### 3.3. In Vitro Metabolic Stability of Violacein in HLMs, MLMs, and RLMs

**Figure 1:**
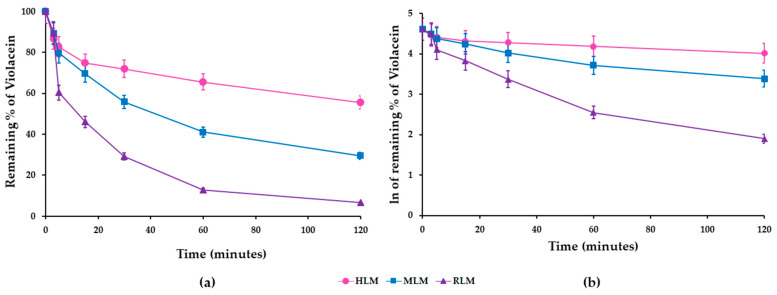
Metabolic stability of violacein in human (HLMs), mouse (MLMs), and rat (RLMs) liver microsomes plotted on a linear scale (**a**) and on a natural logarithm (ln) scale (**b**). The error bars represent the relative standard deviation of the replicate means of three independent experiments.

Metabolic stability was evaluated by linear regression analysis of the residual violacein after incubating 4 µM violacein with 2 mg/mL of HLMs, MLMs, and RLMs in the presence of the enzymatic cofactors required for CYP function in microsomes. Calculation of the in vitro violacein metabolic stability was achieved by plotting the remaining percentage of violacein on the y-axis and the incubation time on the x-axis (Figure 1a). The slope of the linear portion of the natural logarithm (ln) of the remaining percentage of violacein (y-axis) versus incubation time (x-axis) (Figure 1b) corresponded to the metabolism rate constant (k) for violacein (Table 2). The linear regression equation and the coefficient of determination (r^2^) of the linear portion of this graph also provide important information.

**Figure 2:**
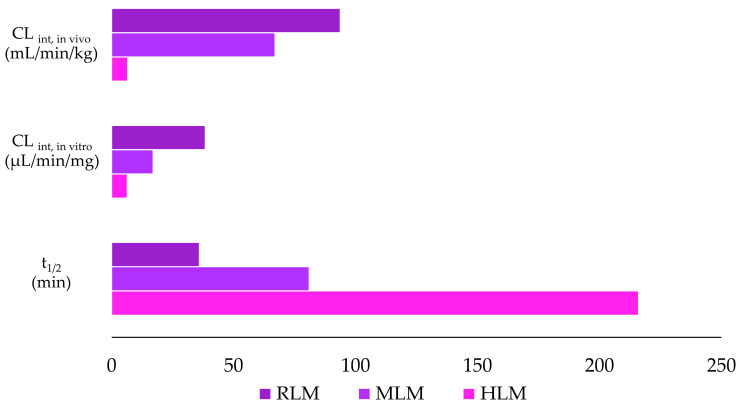
Violacein stability parameters elimination half-life (t_1_/_2_), intrinsic clearance in vitro (CL_int, in vitro_), and intrinsic clearance in vivo (CL_int, in vivo_) in human (HLMs), mouse (MLMs), and rat (RLMs) liver microsomes.

The values for t_1/2_, CL_int, in vitro_, and CL_int, and in vivo_ were calculated for all the microsomal models using the equations described in Section 2.6.1 and are shown in Figure 2 and Table 3.

### 3.4. Identification of Violacein Metabolites in RLMs and HLMs by LC-QTOF

**Figure 3:**
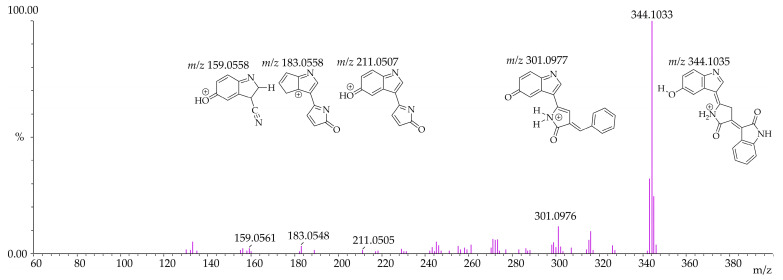
Mass spectrum of violacein in MS/MS positive electrospray ionization (*m*/*z* 344.1033—ESI^+^).

**Figure 4:**
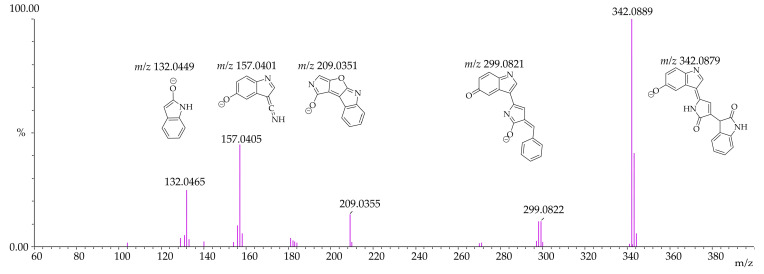
Mass spectrum of violacein in MS/MS negative electrospray ionization (*m*/*z* 342.0889—ESI^−^).

Given that the exact mass of violacein (C_20_H_13_N_3_O_3_) is 343.0956, the expected product ion profile in ESI^+^ mode is the protonated molecule *m*/*z* 344.1035, and in ESI^−^ mode, the deprotonated molecule *m*/*z* 342.0878. Deoxyviolacein (C_20_H_13_N_3_O_2_) has an exact mass of 327.1007, with the expected product ion profile in ESI^+^ mode being the protonated molecule *m*/*z* 328.1086, and in ESI^−^ mode, the deprotonated molecule *m*/*z* 326.0929. Deoxyviolacein was eluted at 13.540 min with *m*/*z* 328.1081 and *m*/*z* 326.0934 in ESI^+^ and ESI^−^ modes, respectively. The mass spectra of violacein in both ESI^+^ and ESI^−^ modes are presented in Figure 3 and Figure 4.

The metabolites were identified according to the criteria described in Section 2.6.2, so that all the metabolites identified corresponded to these parameters, except for the metabolite M3, which displayed a fragment with a mass error >10 ppm (the mass error was 34.58 ppm in ESI^+^ mode and 1.91 ppm in ESI^−^ mode). However, as their precursor ions and fragments were observed in both positive and negative modes, these metabolites were not excluded from the analysis. Even when the mass error of some fragments exceeded that specified, they were found satisfactory when compared to the opposite mode. Since, in addition to the precursor, more than two fragments were required to meet the criterion, even excluding the fragments that did not meet the acceptable mass error threshold, the proposition of this metabolite remained plausible, given that at least two other satisfactory characteristic fragments were present. However, these unsatisfactory fragments were identified and reported because of their potential emergence from the precursor ion.

Four metabolites of violacein were identified in vitro. The M1 (C_26_H_21_N_3_O_9_ *m*/*z* 519.127782) metabolite was the most abundant in both RLM and MLM samples, appearing in samples where only phase II reactions occurred, as well as in those undergoing both phase I and phase II reactions. The formation of M1 was presumably due to the conjugation of violacein with glucuronic acid via the glucuronidation reaction in aromatic alcohols. This reaction involved the O-glucuronidation of the free phenolic hydroxyl group attached to the carbon at position C-6 of the indole ring in the molecule. In RLMs, M1 accounted for approximately 100% of the total metabolites produced through phase II metabolism in samples from phase II reactions, and 48.67% of phase II metabolism in the samples from both phase I and phase II reactions, making it the most abundant for this species. In HLMs, M1 represented 100% of the total metabolites produced through phase II metabolism in samples from phase II reactions, and 70.89% of phase II metabolism in the samples from both phase I and phase II reactions, making it the most abundant for this species. For this assessment, the relative areas obtained in RLMs in ESI^+^ mode were considered. No significant differences in the area obtained in ESI^−^ mode for this species were observed. In HLMs, the relative area values obtained in ESI^−^ mode were analyzed. In ESI^+^ mode, these values were reduced by nearly half.

M2 (C_20_H_15_N_3_O_3_ *m*/*z* 345.1113) is a metabolite derived from reduction, a phase I metabolic reaction thought to occur at the double bond between carbons C-12 and C-17. This metabolite was identified in both RLMs and MLMs. In RLMs, M2 represented approximately 91.84% of the total metabolites produced by phase I metabolism in samples with phase I reactions and 41.69% in samples with both phase I and phase II reactions. In this model, M2 was the second most abundant metabolite. In HLMs, this metabolite represented 75.59% of the total metabolites produced by phase I metabolism in phase I reaction samples and 23.86% in samples containing both phase I and phase II reactions. For this assessment, the relative areas obtained in RLMs analyzed in ESI^+^ mode and in HLMs analyzed in ESI^−^ mode were considered, as these conditions allowed a larger area to be observed for the metabolites formed.

A metabolite was identified that underwent sequential phase I and phase II reactions. M3 (C_26_H_23_N_3_O_9_ *m*/*z* 521.1434) is derived from a reduction, a phase I metabolic reaction proposed to occur at the double bond between carbons C-12 and C-17, followed by a glucuronidation of aromatic alcohols reaction at the free phenolic hydroxyl group attached to the carbon at position C-6 of the indole portion of the molecule. This metabolite was not abundant in HLMs and was only detected in RLMs, accounting for approximately 5.32% of the total metabolites produced by phase II metabolism in the phase II reaction samples. This observation highlights that metabolites generated in phase I and phase II reactions may undergo structural modifications to facilitate conjugation reactions such as glucuronidation. This conclusion was supported by the absence of these metabolites in phase II samples, suggesting that glucuronide conjugation occurred at the same molecular regions that previously underwent phase I reactions. For this assessment, the relative areas obtained in RLMs analyzed in ESI^+^ mode were considered. However, in ESI^−^ mode the relative areas were almost undetectable. In HLMs, ESI^−^ mode was used, while in ESI^+^ mode the relative area was slightly greater, accounting for approximately 1% of the total metabolites under this ionization condition.

The presence of M4 (C_20_H_17_N_3_O_3_ *m*/*z* 347.1269) suggested a double reduction, with the first reduction occurring at the double bond between the C-12 and C-17 carbons, followed by a second reduction at the double bond between the C-13 and C-14 carbons. This metabolite can be identified in both RLMs and MLMs. In RLMs, M4 represented 8.16% of the total metabolites produced through phase I metabolism in phase I reaction samples and 4.32% of the total metabolites produced through phase I metabolism in phase I and phase II reaction samples. In HLMs, this metabolite represented 24.41% of the total metabolites produced through phase I metabolism in phase I reaction samples and 4.99% of the total metabolites produced through phase I metabolism in phase I and phase II reaction samples. For this evaluation, the relative areas obtained in RLMs analyzed in ESI^+^ mode and in HLMs in ESI^−^ mode were considered. However, a significant difference in relative areas was observed in ESI^−^ mode for RLMs, with a pronounced increase under this condition. For RLMs, in ESI^+^ mode this metabolite represented 48.19% of the total metabolites produced through phase I metabolism in phase I reaction samples and 24.43% of the total metabolites produced through phase I metabolism in phase I and phase II reaction samples.

**Figure 5:**
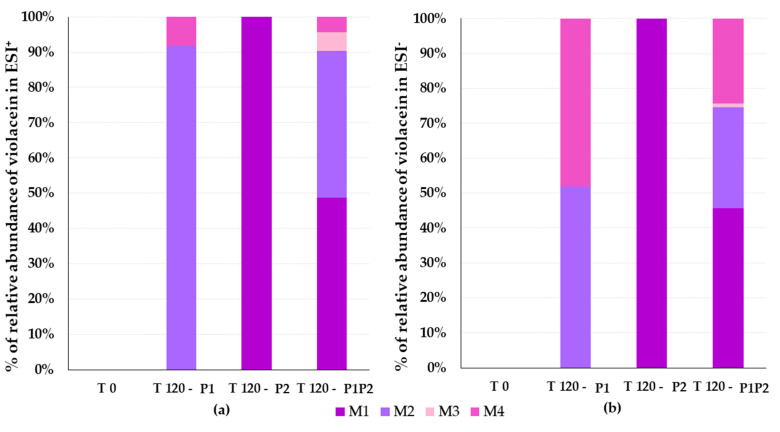
Metabolite formation rates relative to the total area of the identified violacein metabolites in rat liver microsomes (RLMs) considering positive electrospray ionization (ESI^+^) mode (**a**) and negative electrospray ionization (ESI^−^) mode (**b**) in relation to phase I-only reactions (P1), phase II-only reactions (P2), and phase I and phase II reactions (P1P2).

**Figure 6:**
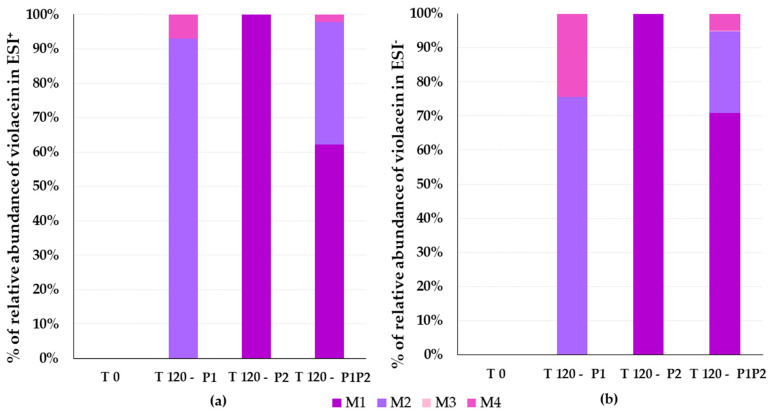
Metabolite formation rates relative to the total area of the identified violacein metabolites in human liver microsomes (HLMs) considering positive electrospray ionization (ESI^+^) mode (**a**) and negative electrospray ionization (ESI^−^) mode (**b**) in relation to phase I-only reactions (P1), phase II-only reactions (P2), and phase I and phase II re-actions (P1P2).

**Figure 7:**
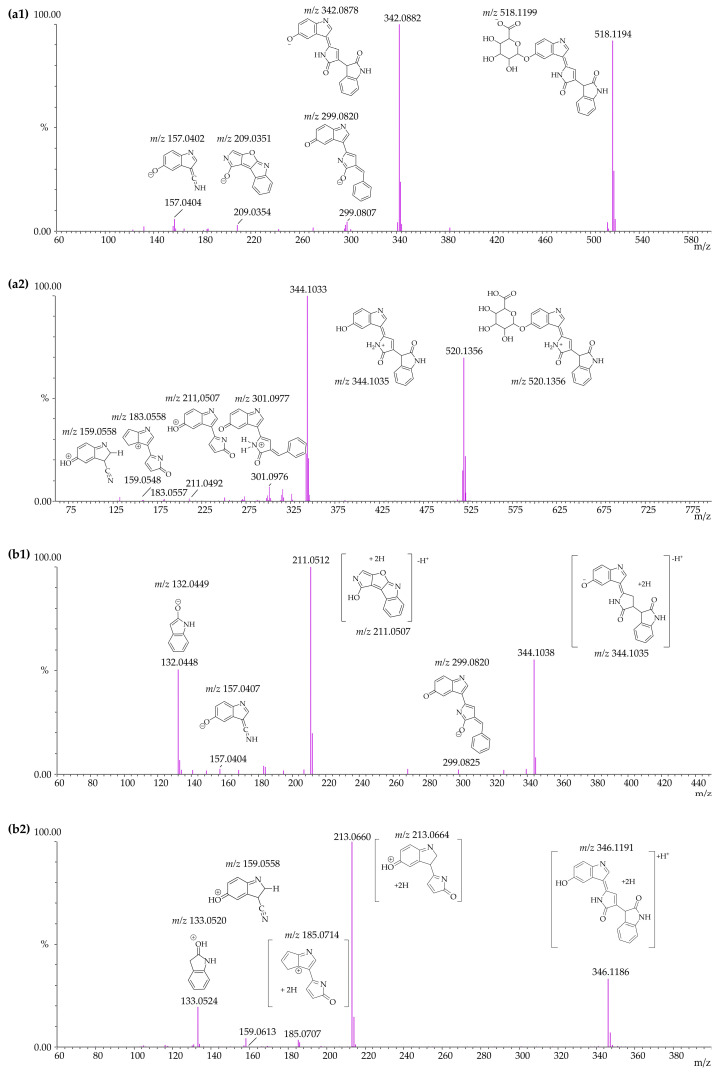

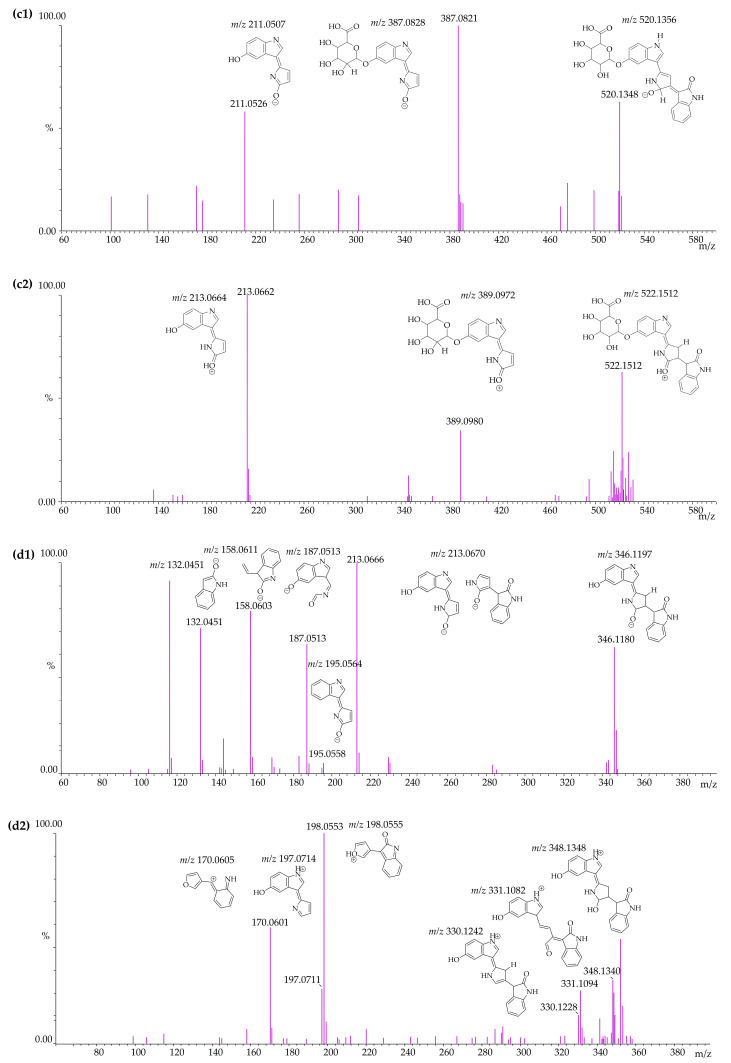
Mass spectrum of M1–violacein–glucuronide in MS/MS negative electrospray ionization (*m*/*z* 518.1194—ESI^−^) mode (**a1**); mass spectrum of M1 in MS/MS positive electrospray ionization (*m*/*z* 520.1356—ESI^+^) mode (**a2**); mass spectrum of M2–violacein–reduced in MS/MS negative electrospray ionization (*m*/*z* 344.1038—ESI^−^) mode (**b1**); and mass spectrum of M2 in MS/MS positive electrospray ionization (*m*/*z* 346.1186—ESI^+^) mode (**b2**); mass spectrum of M3–violacein–reduced–glucoronide in MS/MS negative electrospray ionization (*m*/*z* 520.1348—ESI^−^) mode (**c1**); mass spectrum of M3 in MS/MS positive electrospray ionization (m/*z* 522.1512—ESI^+^) mode (**c2**); mass spectrum of M4–violacein–reduced–reduced in MS/MS negative electrospray ionization (*m*/*z* 346.1180—ESI^−^) mode (**d1**); mass spectrum of M4 in MS/MS positive electrospray ionization (*m*/*z* 348.1340—ESI^+^) mode (**d2**).

The percentages of metabolite formation by HLMs and RLMs were compared using the absolute areas of the chromatographic peaks obtained from the injection of samples prepared as described in Section 2.2.2 and analyzed according to the procedure described in Section 2.6.2. Figure 5 shows the percentages of metabolite formation in RLMs and Figure 6 shows the percentages of metabolite formation in HLMs in relation to phase I-only reactions, phase II-only reactions, and phase I followed by phase II reactions at the end of 120 minutes. Table 4 presents the theoretical mass and measured mass data, and the calculated mass error per fragment for each metabolite. The mass spectra for all metabolites (M1 to M4) in the two ionization modes and their fragments are shown in Figure 7a,b. The fragmentation rationale for each of these metabolites was established and is presented in Figures S3–S5 [63]. The relative percentage of each metabolite was calculated based on the ratio of its chromatographic peak area to the total area of all metabolite peaks obtained from each violacein sample. Approximately 50% of the violacein was metabolized in RLMs, and 30% of the violacein was metabolized in HLMs, in all samples after 120 min of reaction.

## 4. Discussion

The physicochemical characterization of a new drug candidate is crucial for predicting its biological behavior, therapeutic efficacy, and potential challenges in drug development. Computational tools and in vitro assays allow early screening of these properties, helping to optimize the molecule prior to preclinical and clinical evaluation [50]. The ADMET Predictor^®^ estimated a lipophilicity (S + log P) value of 3.226, which aligns with previously reported literature data for violacein. The experimentally determined log P of 2.24 was calculated using a standard curve derived from the log K retention coefficient. Furthermore, ADMET Predictor^®^ microstate pKa simulations yielded values ranging from 11.28 to 10.21, confirming that violacein remains predominantly non-ionized at physiological pH (7.4). The absence of ionization under these conditions underscores the compound’s lipophilic nature, further supported by its log P value [20]. This lipophilicity suggests that violacein falls within the optimal range for passive diffusion across the blood–brain barrier (BBB) and the intestinal epithelium, potentially enhancing its bioavailability [10,20,64]. However, the lack of ionization at physiological pH may also reduce its susceptibility to charge-based interactions, which could influence its pharmacokinetics, cellular uptake, and biological activity [30]. Efforts are being made to improve the physicochemical properties of violacein, particularly its water solubility. Strategies such as nanoparticle formation, use of surfactants, and incorporation of co-surfactants or polymers by sonication have significantly reduced particle size and improved dispersion in water [65].

Comparison of the decreasing areas from the in vitro metabolic stability experiment showed a reduction of the analyte over time (0–120 min), indicating violacein metabolism under the conditions studied. These differences were observed in the graph of remaining percentage of violacein (Figure 1a,b) and in the pharmacokinetic parameters calculated from the results shown in Table 2. At the end of 120 min, approximately 70% of violacein remained in HLMs, compared to around 40% in MLMs and only 10% in RLMs. These findings indicate the high stability (low metabolism) of violacein in HLMs, which is further supported by the pharmacokinetic data obtained. A t_1/2_ of 36 min and CL_int, in vitro_ of 38.4 µL/min/mg for violacein in RLMs indicated a rapid metabolism by these microsomes. A slightly lower rate of metabolism was observed in MLMs, with a t_1/2_ of 81 min and CL_int, in vitro_ of 17.0 µL/min/mg, and much slower metabolism was observed in HLMs, with a t_1/2_ of 216 min and a CL_int, in vitro_ of 6.4 µL/min/mg. These CL_int, in vitro_ values were extrapolated to CL_int, in vivo_ using allometric scaling, and showed similar trends. The CL_int, in vivo_ for RLMs was 93.7 mL/min/kg, indicating rapid metabolism, while MLMs exhibited moderately slower metabolism with a CL_int, in vivo_ of 67.0 mL/min/kg. In contrast, HLMs showed significantly slower metabolism, with a CL_int, in vivo_ of 6.6 mL/min/kg. Human metabolic stability was higher, as indicated by the reduced formation of metabolites over time. An important finding was that violacein showed good chemical stability when exposed to light over a pH range of 5 to 9. Under these conditions, more than 80% of the residual pigment remained unaltered for up to 30 days [65,66].

The clearance and half-life of antimalarial drugs are critical pharmacokinetic parameters for evaluating their efficacy, duration of action, and potential adverse effects in malaria treatment. [67,68]. Since no in vivo or in vitro pharmacokinetic data are currently available for violacein in humans or animal models, a direct comparison between the findings of this study and pre-existing literature data was not feasible. However, the results can be contextualized by drawing parallels with the pharmacokinetic parameters of other antimalarial agents, given violacein’s potential as an antimalarial compound. Drugs such as artemether exhibit a rapid clearance (16.88 ± 4.04 L/h/kg) that is further enhanced when co-administered with pyronaridine (28.00 ± 2.16 L/h/kg) [69], necessitating frequent dosing to maintain therapeutic levels. In contrast, mefloquine (0.02–0.05 L/h/kg) [70] and chloroquine (0.09–0.1 L/h/kg) [71] present significantly lower clearance rates, enabling prolonged dosing intervals and confirming their utility in prophylactic regimens. Quinine, with a clearance of 0.08 to 0.47 L/h/kg [72], presents the potential for drug accumulation, necessitating dose adjustments in patients with hepatic or renal impairment. The estimated CL_int, in vivo_ of primaquine, based on the human hepatic microsomal pathway, is approximately 0.017 mL/min/kg. [73,74,75].

The t_1_/_2_ is an important pharmacokinetic parameter in the development of antimalarial drugs. Malaria treatment depends on factors such as the parasite species, disease duration, and the patient’s clinical condition. Each parasite species may require specific regimens because of varying resistance profiles and responses to antimalarials. The t_1_/_2_ of antimalarials also varies widely among drugs, such as artemisinin (60–120 min), quinine (660–1080 min), lumefantrine (4320–8640 min), and chloroquine (9000 to 17,400 min). This variability affects dosing frequency and treatment duration, enhances efficacy, and minimizes resistance [68,76,77,78].

The multi-species approach revealed significant differences in drug metabolism among humans, rats, and mice, particularly in t_1_/_2_ and CL_int, in vitro_. These findings demonstrate interspecies differences in metabolism, with varying proportions of metabolite formation, and agree with existing literature on interspecies variations in drug metabolism. A comparative study of N-ethyl pentedrone (NEP) metabolism using liver microsomes also revealed a shorter t_1_/_2_ in rats (12.1 min) compared to mice (187 min) and humans (770 min), indicating a faster metabolism in rodents [43]. Midazolam showed a CL_int, in vivo_ of 4.75 L/h/kg in rats compared to 0.40 L/h/kg in humans. Consequently, the t_1_/_2_ of midazolam was about 31 min in rats and 111 min in humans, indicating that the metabolism of this drug occurs significantly faster in rats than in humans [79]. The metabolism of thyroxine (T4) also differs significantly between rats and humans. In rats, the CL_int, in vitro_ was higher (1.08 and 0.75 mL/min/10^6^ cells for rat 1 and 2, respectively) compared to humans (0.56 and 0.61 mL/min/10^6^ cells for donor Hu1362 and 1364, respectively). Consequently, the t_1_/_2_ was shorter in rats (924 and 1332 min) than in humans (1776 and 1614 min) [80]. Humans metabolize certain drugs more slowly than rodents, with this difference reflecting variations in enzyme expression and activity, particularly among CYP450 and UGT enzymes. For example, the CYP3A family exhibits distinct isoforms in humans and rodents, leading to differences in metabolic rates and pathways. Melatonin, derived from tryptophan and structurally similar to violacein, exhibits highly variable metabolism across species. In liver slice studies, the initial rate of metabolism in human samples was approximately half of that observed in rats. This finding suggests lower hepatic extraction in humans, which may lead to higher oral bioavailability compared to rats [81]. The clearance of sumatriptan showed significant variation across species: 2.68 L/h/kg in rats, 1.56 L/h/kg in rabbits, 0.84 L/h/kg in dogs, and approximately 1.10 L/h/kg in humans. These differences reflect variations in metabolic capacity among organisms, as well as tissue-specific expression of monoamine oxidase A (MAO-A), the primary enzyme involved in sumatriptan metabolism. Interestingly, like melatonin and violacein, sumatriptan has a tryptophan-derived structure that may contribute to similarities in its oxidative metabolic pathways [82].

Rodents generally exhibit higher CYP enzyme activity, leading to faster drug elimination, and express CYP3A1, CYP3A2, and CYP3A11, which have different catalytic activities. While CYP1A is conserved across species, its induction by omeprazole occurs only in humans. The CYP2C isoforms vary, with humans expressing CYP2C8, CYP2C9, CYP2C18, and CYP2C19, whereas rodents display sex-related differences. CYP2D6 is polymorphic in humans and shows multiple isoforms in rodents, while CYP3A4, the primary drug-metabolizing enzyme in humans, differs from its rodent counterparts (CYP3A1 in rats, CYP3A11 in mice) [83,84]. Another important difference involves CYP2D6 enzymes. Humans express CYP2D6, which metabolizes antidepressants and β-blockers, while rodents have multiple isoforms such as CYP2D1 and CYP2D2, the activities of which vary with animal sex and strain. Glucuronidation enzymes (UGTs) also differ significantly [83,84]. Among UGTs, humans predominantly express UGT1A and UGT2B, with UGT1A1 playing a critical role in the conjugation of bilirubin and drugs such as irinotecan and morphine. In contrast, UGT2B1 predominates in mice, making data extrapolation challenging because of differences in substrate specificity. Additionally, UGT1A6 is highly expressed in mice, leading to faster metabolism of phenolic compounds compared to humans [83,84,85,86].

In silico analysis identified the formation of four common metabolites predicted by both software tools. Both ADMET Predictor^®^ (version 12.0) and Meteor Nexus (v.3.1.0, Lhasa Limited, Leeds, UK) predicted the formation of phase I metabolites that likely result from benzene hydroxylation (M3 and M11 in ADMET Predictor^®^; M13 and M136 in Meteor Nexus). In addition, phase II metabolites corresponding to the glucuronidation reaction of aromatic alcohols (M1 in ADMET Predictor^®^; M13 in Meteor Nexus) and another resulting from glucuronidation on an aromatic nitrogen (M2 in ADMET Predictor^®^; M19 in Meteor Nexus) were predicted.

In vitro analysis identified the formation of four metabolites: M1 (violacein–glucuronide), M2 (violacein–reduced), M3 (violacein–glucuronide–reduced) and M4 (violacein–reduced–reduced). The phase I metabolic reactions involved in the metabolism of violacein included reduction. For phase II, reactions such as the glucuronidation of aromatic alcohols have been proposed. In the sequential processes of phases I and II, it is proposed that one metabolite undergoes reduction followed by the glucuronidation of aromatic alcohols.

M1 (C_20_H_15_N_3_O_3_ *m*/*z* 345.1113), the most abundant metabolite, resulted from the conjugation of violacein with glucuronic acid via glucuronidation in aromatic alcohols and was identified by both in silico platforms. Meteor Nexus predicted it as M3 (weight 519.1278—score 360) and M4 (weight 519.1278—score 579) and ADMET Predictor^®^ predicted it as M2 (weight 519.127).

M2 (C_20_H_15_N_3_O_3_ *m*/*z* 345.1113), a metabolite derived from the reduction of violacein, was only predicted by Meteor Nexus as M10 (weight 345.1113—score 302), and M3 (C_26_H_23_N_3_O_9_ *m*/*z* 521.1434), a metabolite derived from the reduction of violacein followed by a glucuronidation reaction, was only predicted by Meteor Nexus software as M112 (weight 521.1434—score 175). M4 (C_20_H_17_N_3_O_3_, *m*/*z* 347.1269) is a metabolite resulting from the double reduction of violacein. Notably, this metabolite was not predicted by any of the in silico tools. A complete list of the metabolites predicted by Meteor Nexus and ADMET Predictor^®^ is provided in the Supplementary Materials (Tables S1 and S2).

Both platforms predicted metabolites that were not detected in vitro under the conditions used. The Meteor Nexus in silico platform predicted the formation of a metabolite resulting from a sulfonation reaction (*m*/*z* 424.060334 C_20_H_14_N_3_O_6_S). However, this metabolite was not detected in the spectra of either phase I or combined phase I + II metabolism, in both ESI^+^ and ESI^−^ modes. No peaks corresponding to this metabolite were found in any of the spectra. However, it cannot be ruled out that this metabolite may form in HLMs and RLMs, as the model used in this study was based on hepatic microsomes that predominantly contain enzymes of CYPs and UGTs. Therefore, it is expected that the detected metabolites are primarily of this type. Even with the addition of cofactors, such as UDPGA, PAPS, and SAM, to identify metabolites derived from sulfotransferases and methyltransferases, the chances of success are limited. This is because these enzymes are exclusively located in the cellular cytosol, while hepatic microsomes are predominantly of reticular origin. This represents a limitation of this study. Further studies using liver S9 fraction are necessary to evaluate the formation of this metabolite. ADMET Predictor^®^ identified a hydroxylated metabolite (*m*/*z* 359.0906; C_20_H_13_N_3_O_4_) and a metabolite resulting from a reduction reaction (*m*/*z* 357.0747; C_20_H_11_N_3_O_4_), different from that of the experimentally identified reduced metabolite (*m*/*z* 345.1113; C_20_H_15_N_3_O_3_). However, although both metabolites were investigated by LC-QTOF, their masses were not detected in either ESI^+^ or ESI^−^ mode.

In silico tools played an important role in the initial phase of this investigation by assisting in the analysis of physicochemical parameters and the prediction of potential violacein metabolites. However, as violacein is a novel compound, the use of these tools should be complemented by an exhaustive literature review to ensure a more comprehensive and accurate approach. This combination enables a more robust understanding of the drug’s behavior and minimizes the inherent limitations of computational predictions, thereby ensuring a more reliable analysis. Nevertheless, given that the procedures for assessing metabolic stability are already well established, the in vitro analysis described in this study, combined with the high sensitivity of the analytical techniques used, remains the gold standard. This approach provides essential experimental data to validate and refine computational predictions, ensuring a more precise and reliable assessment of the compound’s metabolism.

Comparison of thein silico and in vitro results confirmed the formation of some previously predicted metabolites, highlighting the importance of integrating these approaches for a more accurate understanding of violacein metabolism. This combination of techniques is crucial in the development of new drugs by allowing a more efficient analysis of the metabolism and toxicological profile of the molecules of interest.

## 5. Conclusions

This study describes the first detailed analysis of the metabolism of violacein. The interspecies evaluation of the metabolic stability of this compound showed a slow elimination profile in HLMs followed by MLMs, and a rapid elimination profile in RLMs, indicating greater resistance to metabolism by HLMs and MLMs when compared with RLMs. Furthermore, four violacein metabolites were identified in HLMs and RLMs, two of which were formed by phase I metabolism, one by phase II metabolism, and one by phase I + II metabolism. The results of this study provide an important basis for future in vitro studies of violacein metabolites, including enzyme induction, inhibition, and kinetic studies. They also provide valuable insights into the toxicity and biological activity of these metabolites and open new opportunities for the development of violacein-based antimalarial drugs.

## Figures and Tables

**Table 1:** Physicochemical properties of violacein predicted in silico by ADMET Predictor^®^.

Parameter Predicted	In Silico	
	Data	Source
log *P*	3.226	ADMET Predictor^®^
Water solubility	0.005 mg/mL	
pKa (strongest base)	11.38	
pKa (strongest acid)	10.21	

**Table 2:** Metabolic stability of violacein in human (HLMs), mouse (MLMs), and rat (RLMs) liver microsomes.

HLMs					
Time	Average Area	RSD (%)	Z (%)	Ln Z	Linear Regression Equation Analytical Parameters
0	1.057	0.3	100	4.605	y = −0.0032x + 4.3894 r^2^ = 0.9651 Slope (k) = 0.0032
3	0.916	0.1	87	4.462	
5 *	0.876	0.0	83	4.417	
15	0.792	0.2	75	4.316	
30	0.762	0.0	72	4.277	
60	0.693	0.1	66	4.183	
120	0.587	0.0	56	4.017	
MLMs					
Time	Average Area	RSD (%)	Z (%)	Ln Z	Linear Regression Equation Analytical Parameters
0	0.796	0.0	100	4.605	y = −0.0085x + 4.3405 r^2^ = 0.9554 Slope (k) = 0.0085
3	0.710	0.1	89	4.490	
5 *	0.635	0.0	80	4.379	
15	0.554	0.0	70	4.244	
30	0.444	0.0	56	4.022	
60	0.327	0.0	41	3.715	
120	0.235	0.0	30	3.385	
RLMs					
Time	Average Area	RSD (%)	Z (%)	Ln Z	Linear Regression Equation Analytical Parameters
0	1.333	0.0	100	4.605	
3	1.193	0.8	90	4.495	y = −0.0192x + 4.0344 r^2^ = 0.9459 Slope (k) = 0.0192
5 *	0.805	0.4	60	4.101	
15	0.615	0.1	46	3.832	
30	0.388	0.1	29	3.371	
60	0.171	0.1	13	2.549	
120	0.089	0.1	7	1.899	

* The linear graph representing the remaining percentage of violacein was constructed using triplicate data, with the values highlighted in bold; RSD, the relative standard deviation; Z, the remaining percentage of violacein.

**Table 3:** In vitro elimination half-life (t_1_/_2_), intrinsic clearance in vitro (CL_int, in vitro_), and intrinsic clearance in vivo (CL_int, in vivo_) for violacein in human (HLMs), mouse (MLMs), and rat (RLMs) liver microsomes.

Parameter	Species		
	Human	Mouse	Rat
t_1/2_ (min)	216	81	36
CL_int, in vitro_ (µL/min/mg)	6.40	17.01	38.41
CL_int, in vivo_ (mL/min/kg)	6.58	66.99	93.72

Data expressed as means (*n* = 3).

**Table 4:** Violacein and metabolite data obtained in rat (RLMs) and human (HLMs) liver microsomes.

Microsomal Model	Metabolism Reaction	Fragment	Molecular Formula Neutral	Exact Mass (ppm)	Theoretical Exact Mass [M + H]^+^	Measured Exact Mass [M + H]^+^	Mass Error (ppm)	Theoretical Exact Mass [M − H]^−^	Measured Exact Mass [M − H]^−^	Mass Error (ppm)	Retention Time (min)
Violacein		-	C_20_H_13_N_3_O_3_	343.0956	344.1035	344.1033	0.63	342.0878	342.0889	−3.02	[M + H]^+^ 12.15 [M − H]^−^ 12.14
RLMs HLMs	Violacein -	F1	C_19_H_12_N_2_O_2_	300.0898	301.0977	301.0976	0.34	299.0821	299.0822	−0.49	
		F2	C_12_H_6_N_2_O_2_	210.0429	211.0507	211.0505	0.95	209.0351	209.0355	−1.91	
		F3	C_11_H_6_N_2_O	182.0480	183.0558	183.0548	5.67	-	-	-	
		F4	C_9_H_6_N_2_O	158.0480	159.0558	159.0561	−1.65	157.0401	157.0405	−2.55	
		F5	C_8_H_7_NO	133.0527	-	-	-	132.0449	132.0465	−12.12	
Violacein–glucuronide		M1	C_26_H_21_N_3_O_9_	519.1277	520.1356	520.1356	0.00	518.1199	518.1194	0.97	[M + H]^+^ 10.10 and 10.85 [M − H]^−^ 10.42 and 10.86
RLMs HLMs	Glucuronidation	F6	C_20_H_13_N_3_O_3_	343.0957	344.1035	344.1033	0.58	342.0878	342.0882	−1.17	
		F1	C_19_H_12_N_2_O_2_	300.0899	301.0977	301.0976	0.33	299.0820	299.0807	4.35	
		F2	C_12_H_6_N_2_O_2_	210.0429	211.0507	211.0492	7.11	209.0351	209.0354	−1.44	
		F3	C_11_H_6_N_2_O	182.0480	183.0558	183.0557	0.55	-	-	-	
		F4	C_9_H_6_N_2_O	158.0480	159.0558	159.0548	6.29	157.0402	157.0404	−1.35	
Violacein–reduced		M2	C_20_H_15_N_3_O_3_	345.1113	346.1191	346.1186	1.44	344.1035	344.1038	−0.82	[M + H]^+^ 7.41 and 7.84 [M − H]^−^ 7.39 and 7.83
RLMs HLMs	Reduction	F1	C_19_H_12_N_2_O_2_	300.0898	-	-	-	299.0820	299.0825	−1.67	
		F7	C_12_H_8_N_2_O_2_	212.0585	213.0664	213.066	1.88	211.0507	211.0512	−2.37	
		F8	C_11_H_8_N_2_O	184.0636	185.0714	185.0707	3.78	-	-	-	
		F4	C_9_H_6_N_2_O	158.0480	159.0558	159.0613	−34.58 *	157.0401	157.0404	−1.91	
		F9	C_8_H_6_NO	133.0527	133.0520	133.0524	−3.01	132.0449	132.0448	0.76	
Violacein reduced glucuronide		M3	C_26_H_23_N_3_O_9_	521.1434	522.1512	522.1512	0.00	520.1356	520.1348	1.54	[M + H]^+^ 5.13 and 6.25 [M − H]^−^ 5.04 and 6.25
RLMs	Reduction + glucuronidation	F10	C_18_H_16_N_2_O_8_	388.0906	389.0984	389.0972	3.08	387.0828	387.0821	1.81	
		F7	C_12_H_8_N_2_O_2_	212.0585	213.0664	213.0662	0.94	211.0507	211.0526	−9.00	
Violacein–reduced–reduced		M4	C_20_H_17_N_3_O_3_	347.1269	348.1348	348.1340	2.30	346.1191	346.1180	3.18	[M + H]^+^ 12.15 [M − H]^−^ 12.14
RLMs HLMs	Reduction + reduction	F11	C_20_H_16_N_2_O_3_	332.1160	331.1082	331.1094	−3.62	-	-	-	
		F12	C_20_H_17_N_3_O_2_	331.1320	330.1242	330.1228	4.24	-	-	-	
		F13	C_12_H_10_N_2_O_2_	214.0742	-	-	-	213.0664	213.0666	−0.94	
		F14	C_12_H_9_NO_2_	199.0633	198.0555	198.0553	1.01	-	-	-	
		F15	C_12_H_10_N_2_O	198.0793	197.0714	197.0711	1.52	-	-	-	
		F16	C_12_H_10_N_2_O_2_	196.0636	-	-	-	195.0558	195.0558	0.00	
		F17	C_10_H_8_N_2_O_2_	188.0585	-	-	-	187.0507	187.0513	−3.21	
		F18	C_11_H_8_NO	171.0684	170.0605	170.0601	2.35	-	-	-	

* Identification of a metabolite with a fragment showing a mass error >10 ppm. Since precursor ions and fragments were detected in both positive and negative modes, the metabolite was not excluded. Despite some fragments exceeding the acceptable mass error threshold, the identification remained valid as at least two other fragments met the criteria. Unsatisfactory fragments were reported because of their potential origin from the precursor ion.

## Data Availability

The data presented in this study are available in this article.

## Supplementary Materials

The following supporting information can be downloaded at https://www.mdpi.com/article/10.3390/pharmaceutics17050601/s1, Figure S1: Microstate pKa analysis by MedChem Designer^®^; Table S1: In silico metabolites predicted by Meteor Nexus; Table S2: In silico metabolites predicted by ADMET Predictor^®^; Figure S2: Chemical structures of metabolites resulting from predictions made by ADMET Predictor^®^; Figure S3: Violacein fragmentation pathway—*m*/*z* 344.103517— positive electrospray ionization mode—MS/MS—pathways I to V, and *m*/*z* 342.0889—negative electrospray ionization mode—MS/MS—pathways I to III; Figure S4: Violacein–reduced–glucoronide fragmentation pathway—M3—positive electrospray ionization mode—MS/MS, and negative electrospray ionization mode—MS/MS; Figure S5: Violacein–reduced–reduced fragmentation pathway—M4—negative electrospray ionization mode—MS/MS—pathways I and II, and positive electrospray ionization mode—MS/MS—pathway I to III.
