# Supplementary material for: Evaluation of Violacein Metabolic Stability and Metabolite Identification in Human, Mouse, and Rat Liver Microsomes
Source: Pharmaceutics. 2025 May 2;17(5):601. doi: 10.3390/pharmaceutics17050601 (PMC12114947; doi:10.3390/pharmaceutics17050601)
Supplement: Supplementary file 1 [file pharmaceutics-17-00601-s001.zip › pharmaceutics-3532277-supplementary.pdf]

pharmaceutics

MDPI

Supplementary Material

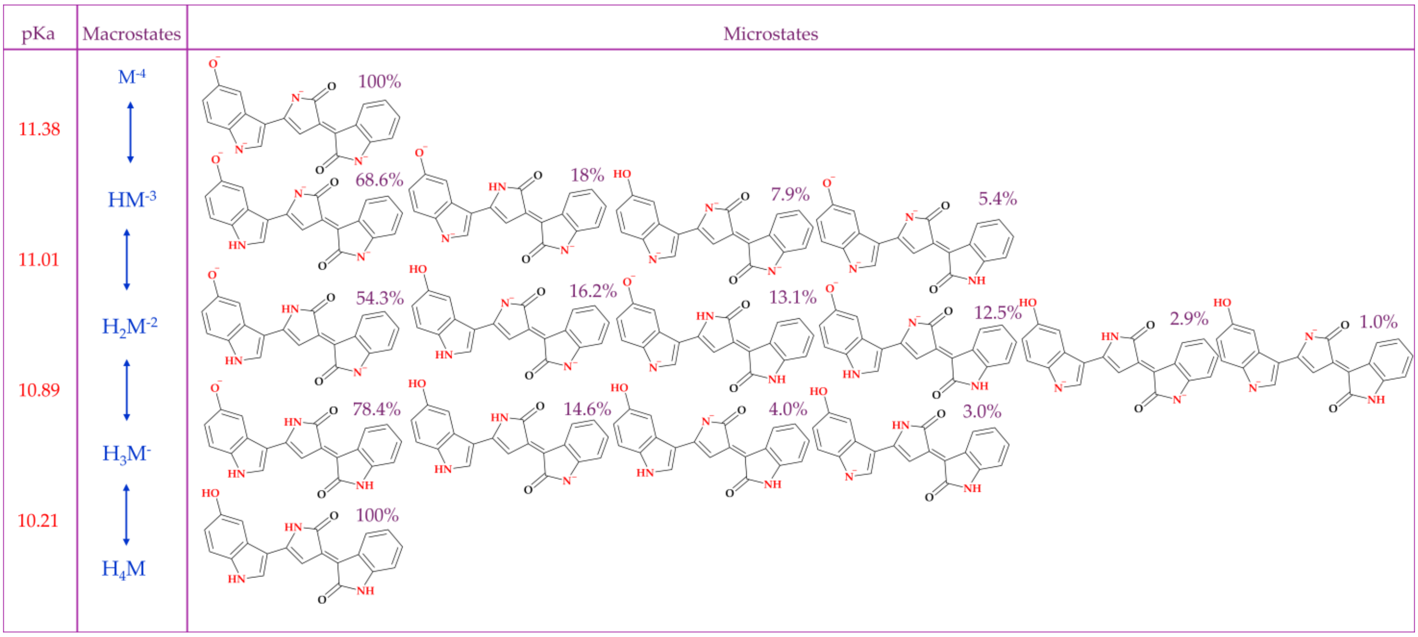

Figure S1. Microstate pKa analysis by MedChem Designer®.

Table S1. In silico metabolites predicted by Meteor Nexus.

Metabolite	Weight	Score	Type/Reaction
M1	423.0525	242	O-Sulphonation of aromatic alcohols
M2	423.0525	121	O-Sulphonation of aromatic alcohols
M3	519.1278	360	Glucuronidation of aromatic alcohols
M4	519.1278	579	Glucuronidation of aromatic alcohols
M10	345.1113	302	Reduction of alpha,beta-unsaturated compounds
M11	361.1063	85	Hydrolysis of Imines
M13	359.0906	235	5-Hydroxylation of 1,2,4-trisubstituted benzenes
M18	505.1485	109	Glucosidation of alcohols
M19	519.1258	109	Glucuronidation at aromatic Nitrogen
M24	599.0846	192	O-Sulphonation of aromatic alcohols (M1)<br>Glucuronidation of aromatic alcohols
M64	695.1599	174	Glucuronidation of aromatic alcohols (M3)<br>Glucuronidation of aromatic alcohols
M64	695.1599	151	Glucuronidation of aromatic alcohols (M4)<br>Glucuronidation of aromatic aalcohols
M79	535.1227	105	5-Hydroxylation of 1,2,4-trisubstituted benzenes (M13)<br>Glucuronidation of aromatic alcohols
M91	695.1599	153	Glucuronidation of aromatic alcohols (M3)<br>Glucuronidation at aromatic nitrogen
M112*	521.1434	175	Reduction of alpha,beta-unsaturated compounds (M10)<br>Glucuronidation of aromatic alcohols
M112	512.1434	71	Glucuronidation of aromatic alcohols (M4)<br>Reduction of alpha,beta-unsaturated compounds
M125	695.1599	244	Glucuronidation of aromatic alcohols (M4)<br>Glucuronidation at aromatic nitrogen
M136	361.1063	71	5-Hydroxylation of 1,2,4-trisubstituted benzenes (M13)<br>Reduction of alpha,beta-unsaturated compounds
M136	361.1063	71	Reduction of alpha,beta-unsaturated compounds (M10)<br>5-Hydroxylation of 1,2,4-trisubstituted benzenes
M167	535.1227	119	5-Hydroxylation of 1,2,4-trisubstituted benzenes (M13)<br>Glucuronidation of aromatic alcohols
M168	535.1227	118	5-Hydroxylation of 1,2,4-trisubstituted benzenes (M13)<br>Glucuronidation of aromatic alcohols
M269	775.1167	120	O-Sulphonation of aromatic alcohols (M1)<br>Glucuronidation of aromatic alcohols (M24)<br>Glucuronidation at aromatic nitrogen
M406	697.1755	74	Reduction of alpha,beta-unsaturated compounds (M10)<br>Glucuronidation of aromatic alcohols (M112)<br>Glucuronidation at aromatic nitrogen
M422	871.192	91	Glucuronidation of aromatic alcohols (M4)<br>Glucuronidation at aromatic nitrogen (M125)<br>Glucuronidation of carboxylic acids
M423	871.192	80	Glucuronidation of Aromatic Alcohols (M4)<br>Glucuronidation at Aromatic Nitrogen (M125)<br>Glucuronidation of Carboxylic Acids

* Metabolite M112 -Meteor Nexus, formed from the reduced metabolite M10 -Meteor Nexus, highlighting the dependence of the phase II reaction on the prior occurrence of a phase I reaction. Common metabolite with in vitro experiment (M3).

Table S2. In silico metabolites predicted by ADMET Predictor®.

Metabolite	Weight	Reaction	Enzyme
M1	359.343	5-Hydroxylation of 1,2,4-Trisubstituted Benzenes	CYP1A2;CYP2C9
M2	357.327	Reduction of Benzenes	CYP1A2;CYP2C9
M3	359.343	5-Hydroxylation of 1,2,4-Trisubstituted Benzenes	CYP1A2;CYP2C9;CYP3A4
M4	357.327	Reduction of Benzenes	CYP1A2;CYP2C9;CYP3A4
M5	359.343	5-Hydroxylation of 1,2,4-Trisubstituted Benzenes	CYP1A2;CYP2C9;CYP3A4
M6	357.327	Reduction of Benzenes	CYP1A2;CYP2C9;CYP3A4
M7	359.343	5-Hydroxylation of 1,2,4-Trisubstituted Benzenes	CYP2C9
M8	359.343	5-Hydroxylation of 1,2,4-Trisubstituted Benzenes	CYP3A4
M9	359.343	5-Hydroxylation of 1,2,4-Trisubstituted Benzenes	CYP3A4
M10	359.343	5-Hydroxylation of 1,2,4-Trisubstituted Benzenes	CYP1A2;CYP2C9;CYP3A4
M11	359.343	5-Hydroxylation of 1,2,4-Trisubstituted Benzenes	CYP1A2;CYP2C9;CYP3A4
M12	359.343	5-Hydroxylation of 1,2,4-Trisubstituted Benzenes	CYP1A2;CYP2C9;CYP3A4
M1	519.127	Glucuronidation of Aromatic Alcohols	UGT1A1;UGT1A6;UGT1A8;UGT1A9
M2	519.127	Glucuronidation at Aromatic Nitrogen	UGT1A10

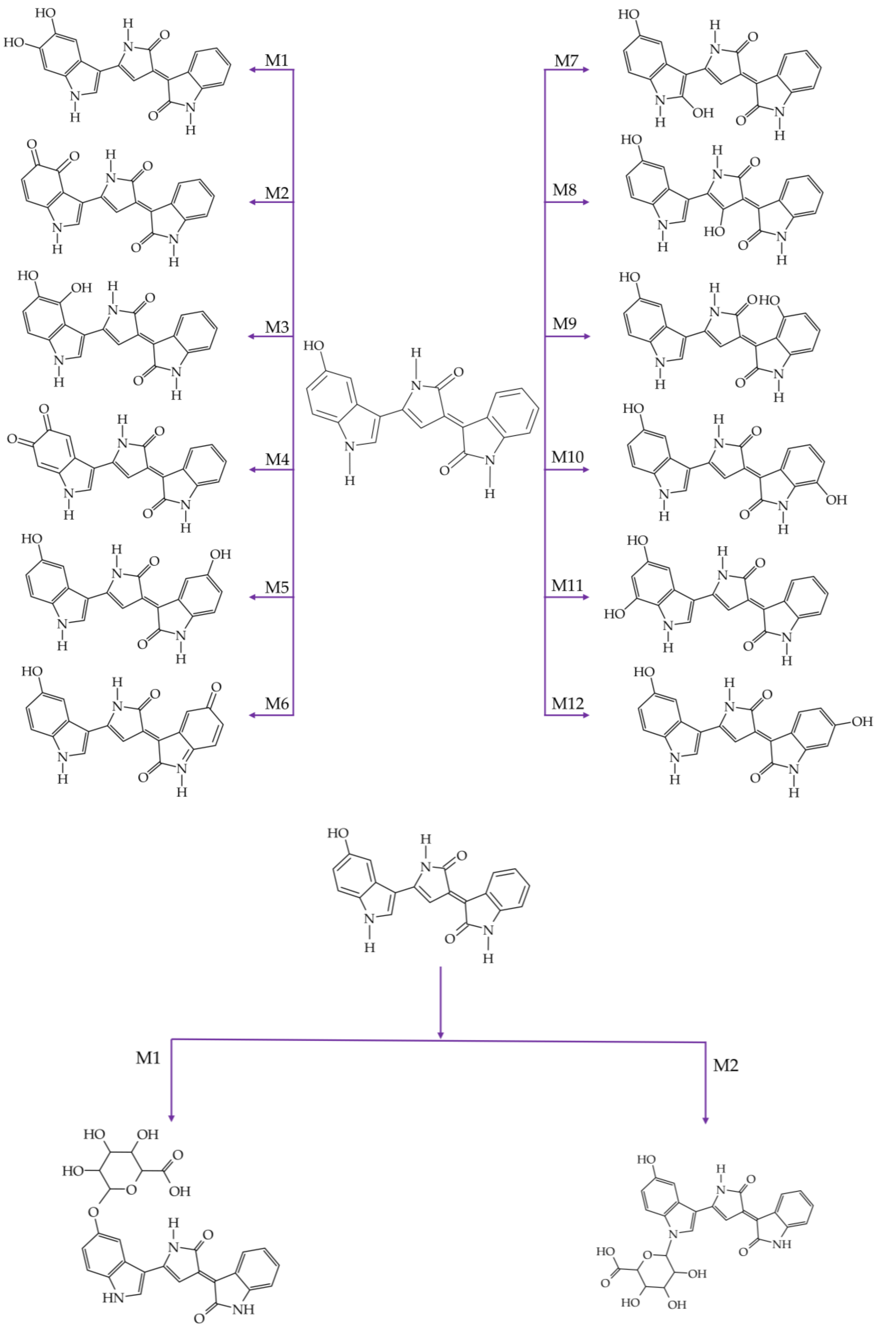

Figure S2. Chemical structures of metabolites result from predictions made by the ADMET Predictor®.

Pathway I: By CO elimination from the amide cyclic system (Item 4.8 - Nat. Prod. Rep. 2016, 33, 432-455) via the formation of an acylium ion, followed by NH_3 loss through a charge migration displacement reaction (Item 7.1.3 - Nat. Prod. Rep. 2016, 33, 432-455), and finally, CO elimination from carbonyls in cyclic systems.

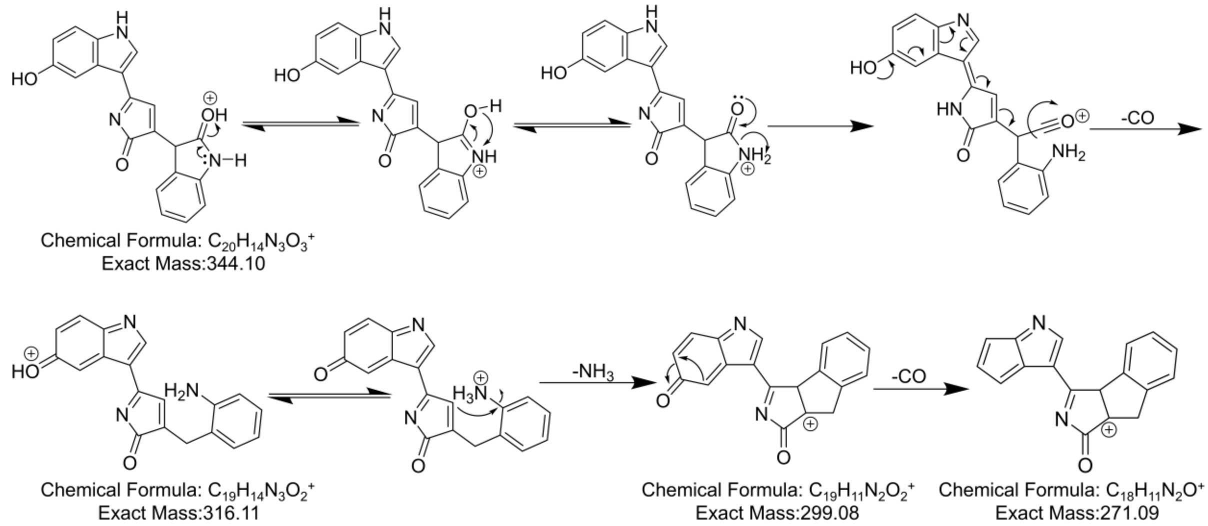

Pathway II: Formation of m/z 316, 299, and 159 ions by CO elimination from the amide cyclic system (Item 4.8 - Nat. Prod. Rep. 2016, 33, 432-455) via the formation of an acylium ion, followed by NH_3 loss through a charge migration displacement reaction (Item 7.1.3 - Nat. Prod. Rep. 2016, 33, 432-455), and a parallel fragmentation of the m/z 316 ion via a charge-retention mechanism through a retro-ene reaction (Item 4.3 - Nat. Prod. Rep. 2016, 33, 432-455).

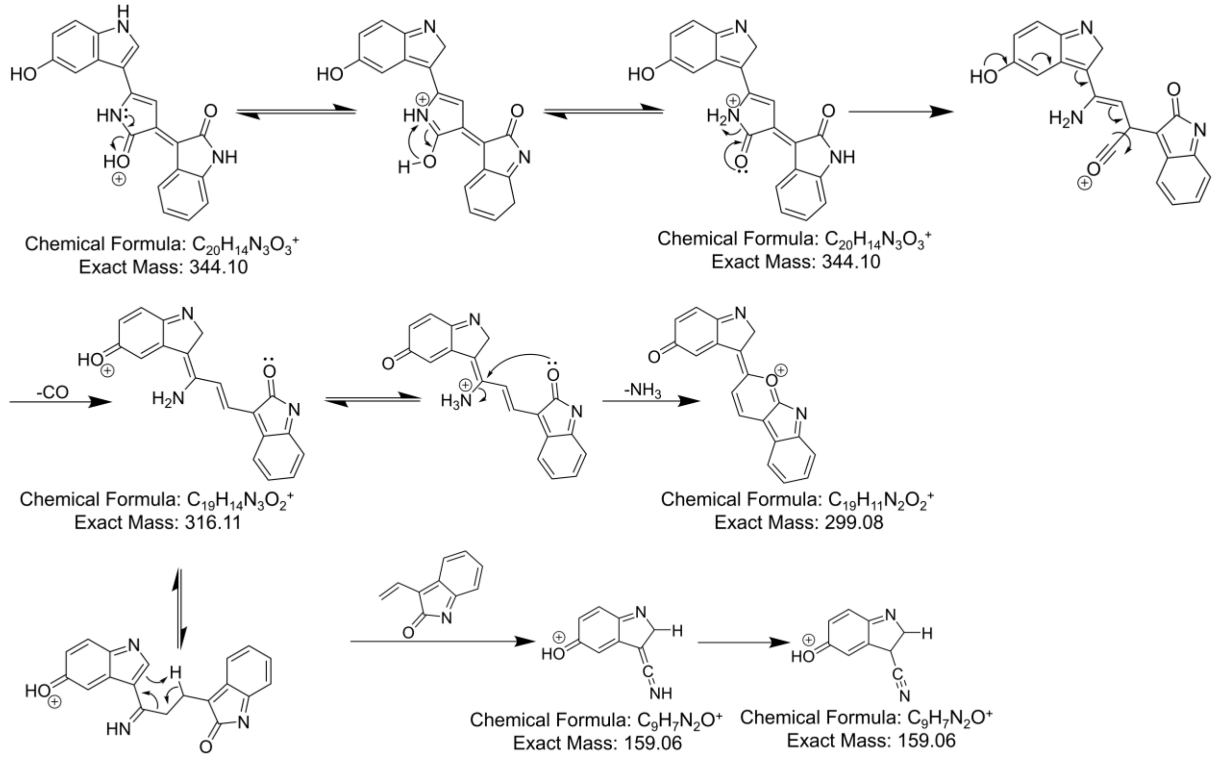

Figure S3. Violacein fragmentation pathway – *m/z* 344.103517– Positive electrospray ionization mode – MS/MS - Pathway I and Pathway II.

Pathway III: Formation of *m*/*z* 326 and 298 ions by protonation at the pentacyclic amide site, followed by a sigmatropic hydrogen rearrangement stabilized by resonance, H_2O elimination, and finally, a neutral CO loss.

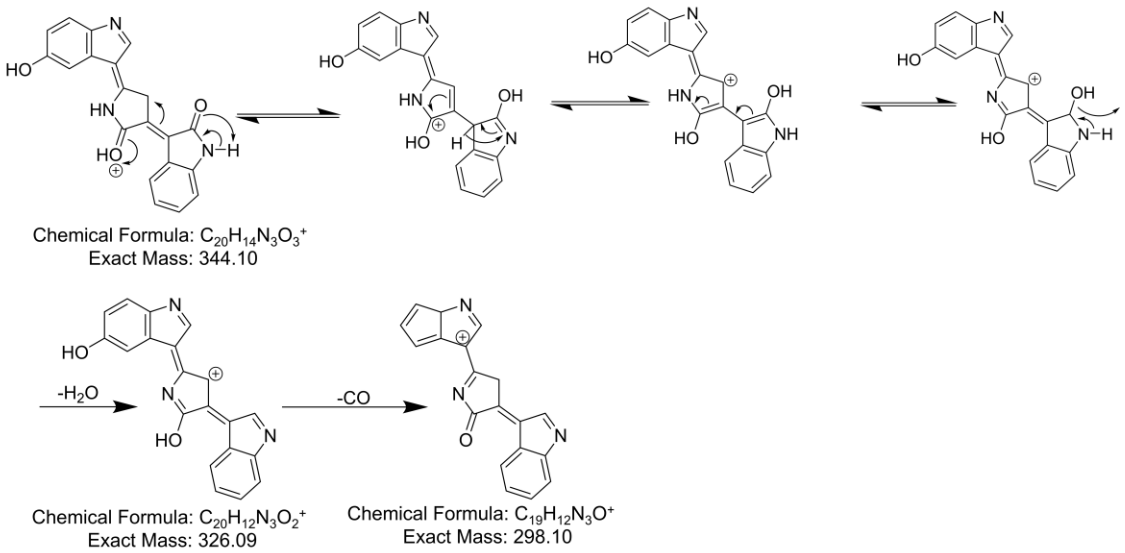

Pathway IV: Two routes for the formation of *m*/*z* 301 and 273 ions by remote hydrogen rearrangement and isocyanic acid elimination, followed by carbon monoxide loss.

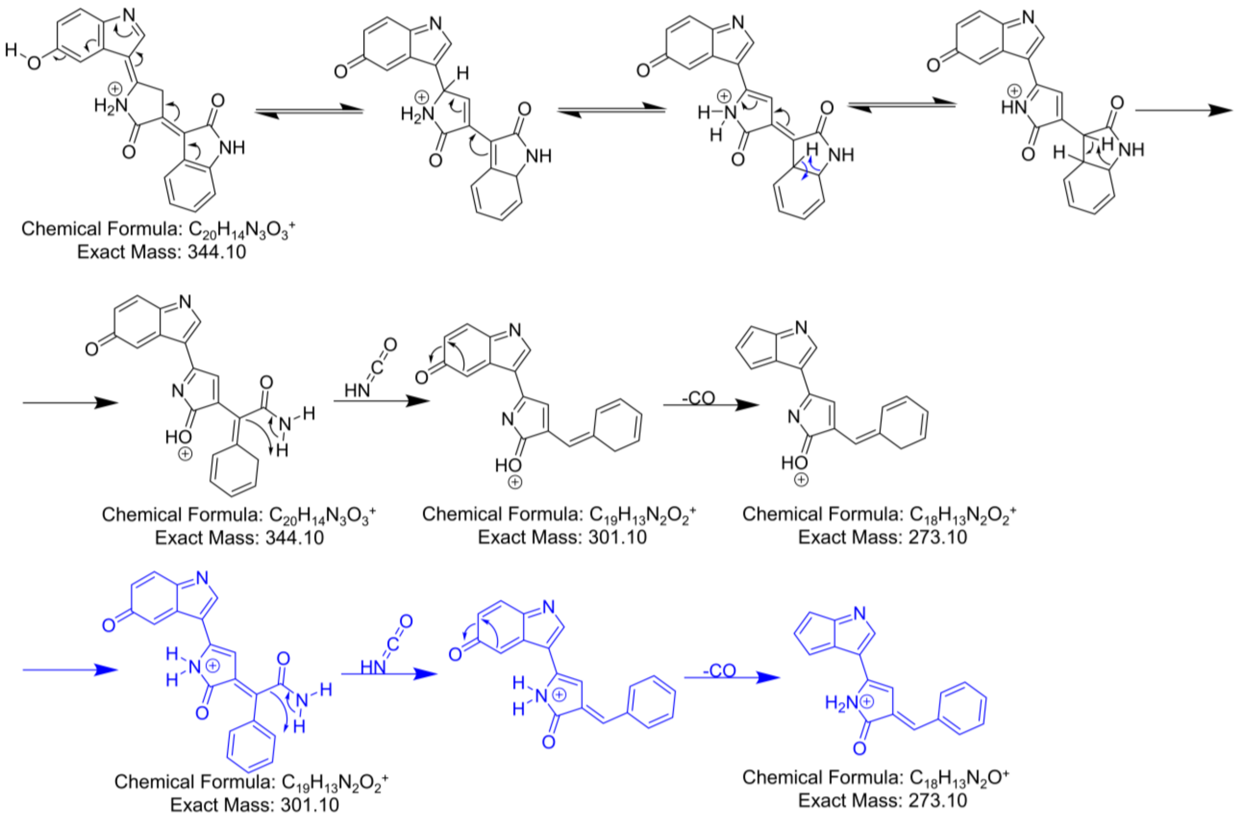

Figure S3 Continued. Violacein fragmentation pathway – *m*/*z* 344.103517– Positive electrospray ionization mode – MS/MS - Pathway III and Pathway IV.

Pathway V: Formation of m/z 211 and 183 ions, initiated by conjugation of the violacein aromatic system, followed by an elimination reaction via retro-heteroene, and finally, a neutral CO loss

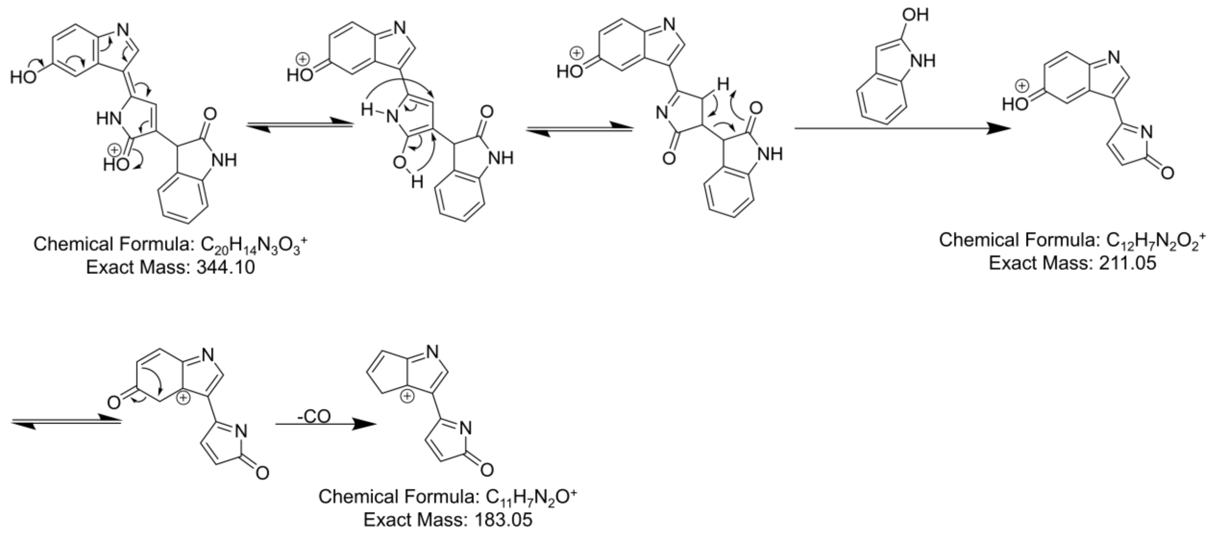

Figure S3 Continued. Violacein fragmentation pathway – *m*/*z* 344.103517– Positive electrospray ionization mode – MS/MS - Pathway V.

Pathway I: Conjugation to a 1,4-quinone methide form, leaving the negative charge on the carbonyl group of the pentacyclic amide ring. In blue, we highlight the formation of ion *m*/*z* 299.0826 by a remote hydrogen rearrangement, followed by isocyanic acid elimination and a neutral loss of CO (*m*/*z* 271.0877) from the quinone methide system via cyclic carbonyl group. In parallel (in black), the resonance system extends to the carbonyl group of the indole system, restoring aromaticity in the six-membered ring and leading to the neutral loss of $C_{12}H_6N_2O_2$ via remote hydrogen rearrangement, resulting in the product ion *m*/*z* 132.0455.

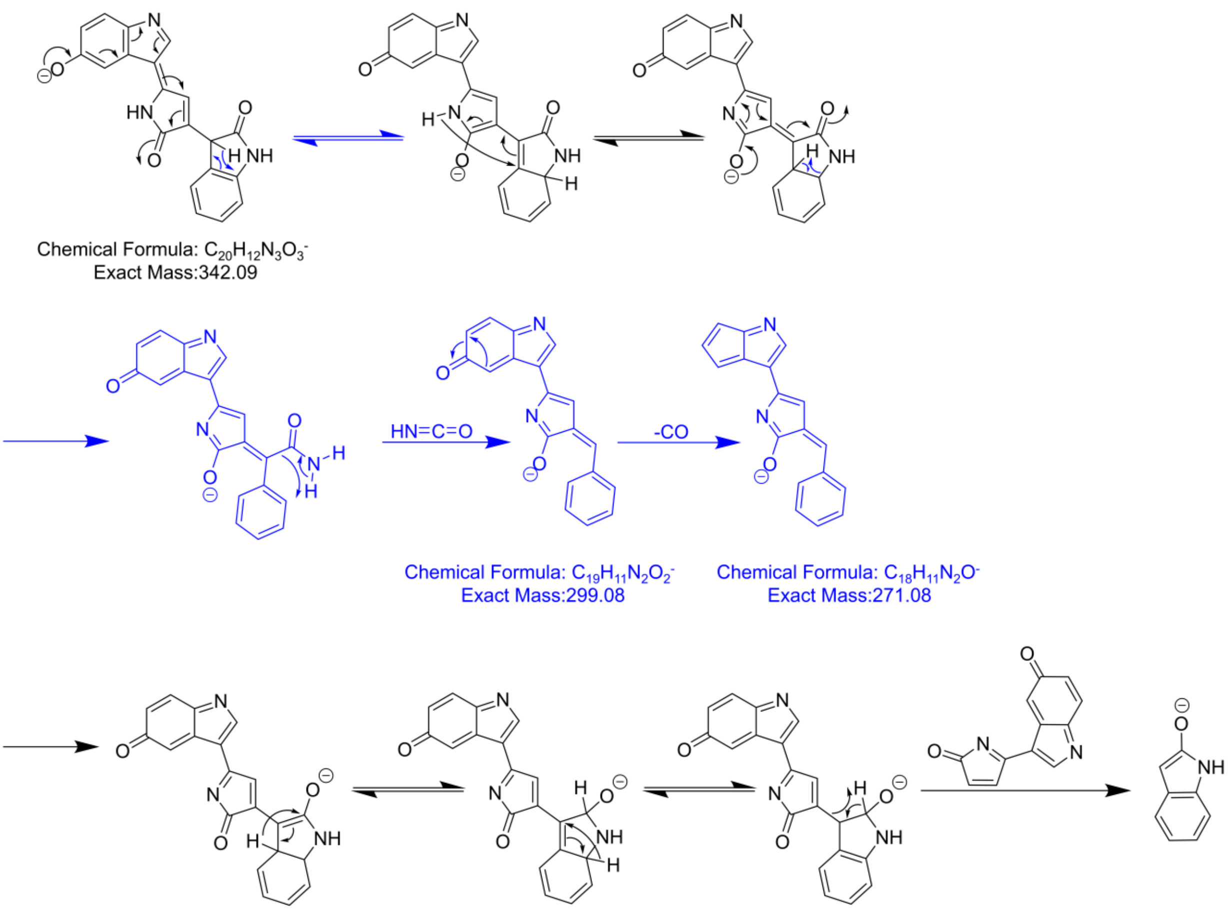

Pathway II: The formation of ion *m*/*z* 209.0357 begins with a displacement reaction from the deprotonated carbonyl group towards the β-carbon of the α,β-unsaturated amide system, leading to the formation of a furanic derivative. This is followed by a remote hydrogen rearrangement, resulting in the loss of C_8H_7NO.

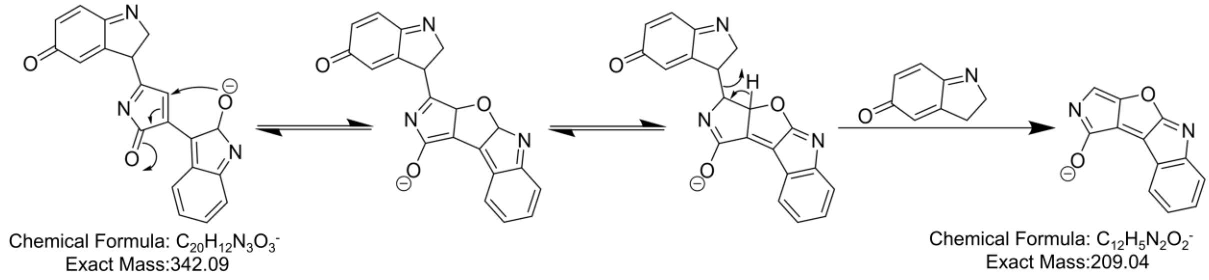

Figure S3 Continued. Violacein fragmentation pathway – *m*/*z* 342.0889– Negative electrospray ionization mode – MS/MS - Pathway I and Pathway II.

Pathway III: The formation of ion *m/z* 157.0407 begins with the conjugation of the amide system to the 1,4-quinone methide system, followed by a sequential sigmatropic rearrangement leading to the formation of an enolate, which then undergoes a γ-elimination, resulting in the neutral loss of $C_{11}H_7NO_2$.

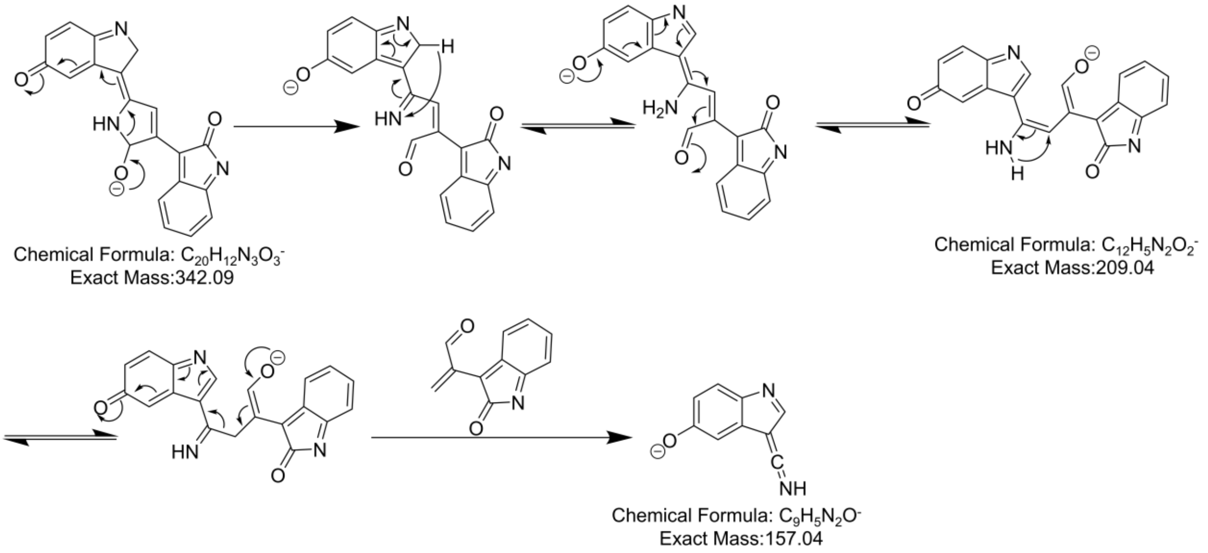

Figure S3 Continued. Violacein fragmentation pathway – *m/z* 342.0889– Negative electrospray ionization mode – MS/MS - Pathway III.

Pathway I: The formation of ion *m*/*z* 387 begins with sequential hydrogen sigmatropic rearrangements, followed by a retro-heteroene reaction resulting in a neutral loss of C_8H_7NO. The last step involves the cleavage of the glucuronide moiety, leading to the formation of ion m/z 211.

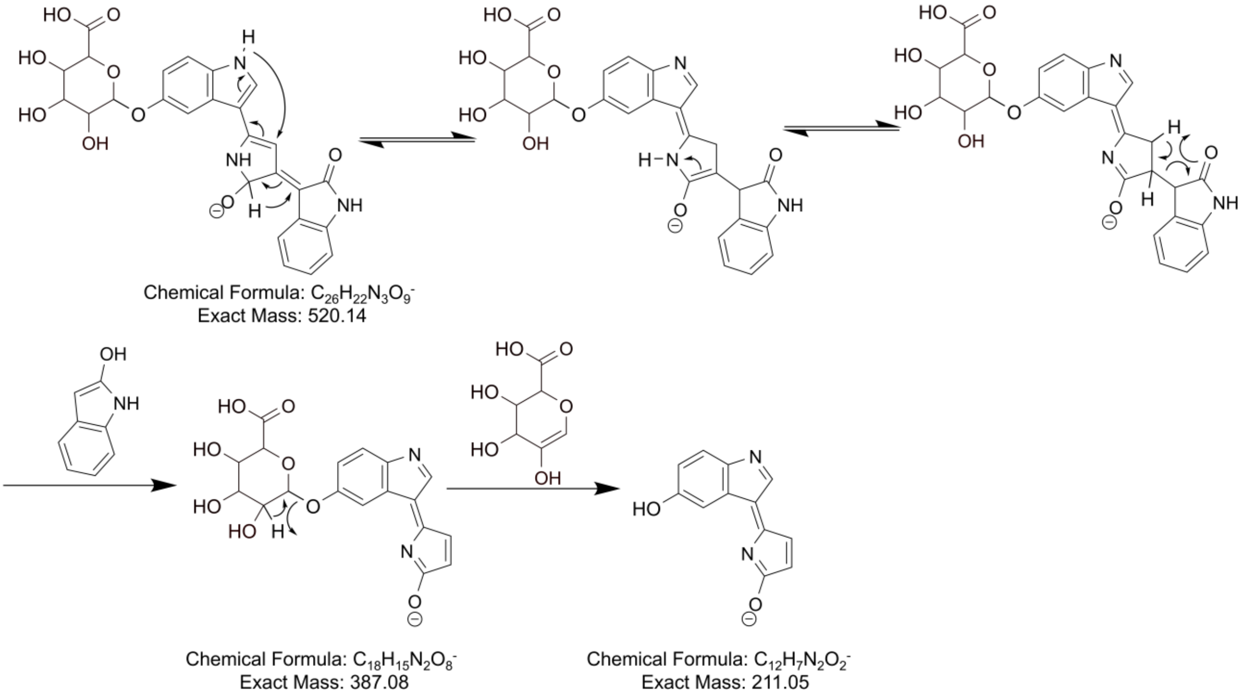

Figure S4. Violacein-Reduced-Glucoronide fragmentation pathway – M3– Negative mode – MSMS

Pathway I: The formation of ion *m*/*z* 389 begins by a retro-heteroene reaction resulting in a neutral loss of C_8H_7NO followed by the cleavage of the glucuronide moiety, leading to the formation of ion m/z 213**.**

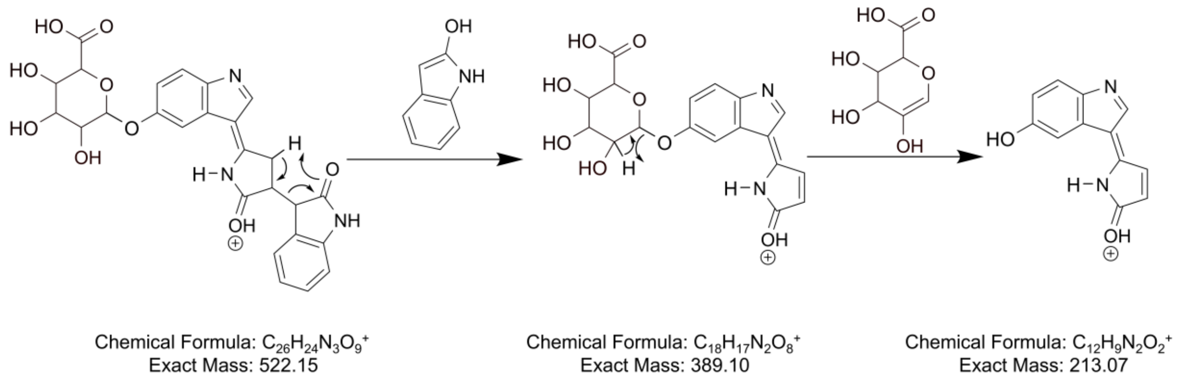

Figure S4. Violacein-Reduced-Glucoronide fragmentation pathway – M3 – Positive electrospray ionization mode – MS/MS.

Pathway I: The formation of ion *m*/*z* 213 occurs through a retro-heteroene elimination reaction, followed by a neutral loss of water, resulting in *m*/*z* 195.In parallel, ion *m*/*z* 346 undergoes a remote hydrogen rearrangement, leading to the elimination of C_8H_7NO and the formation of fragment *m*/*z* 213, which subsequently undergoes a sequential neutral loss of C_4H_3NO, generating ion *m*/*z* 132.

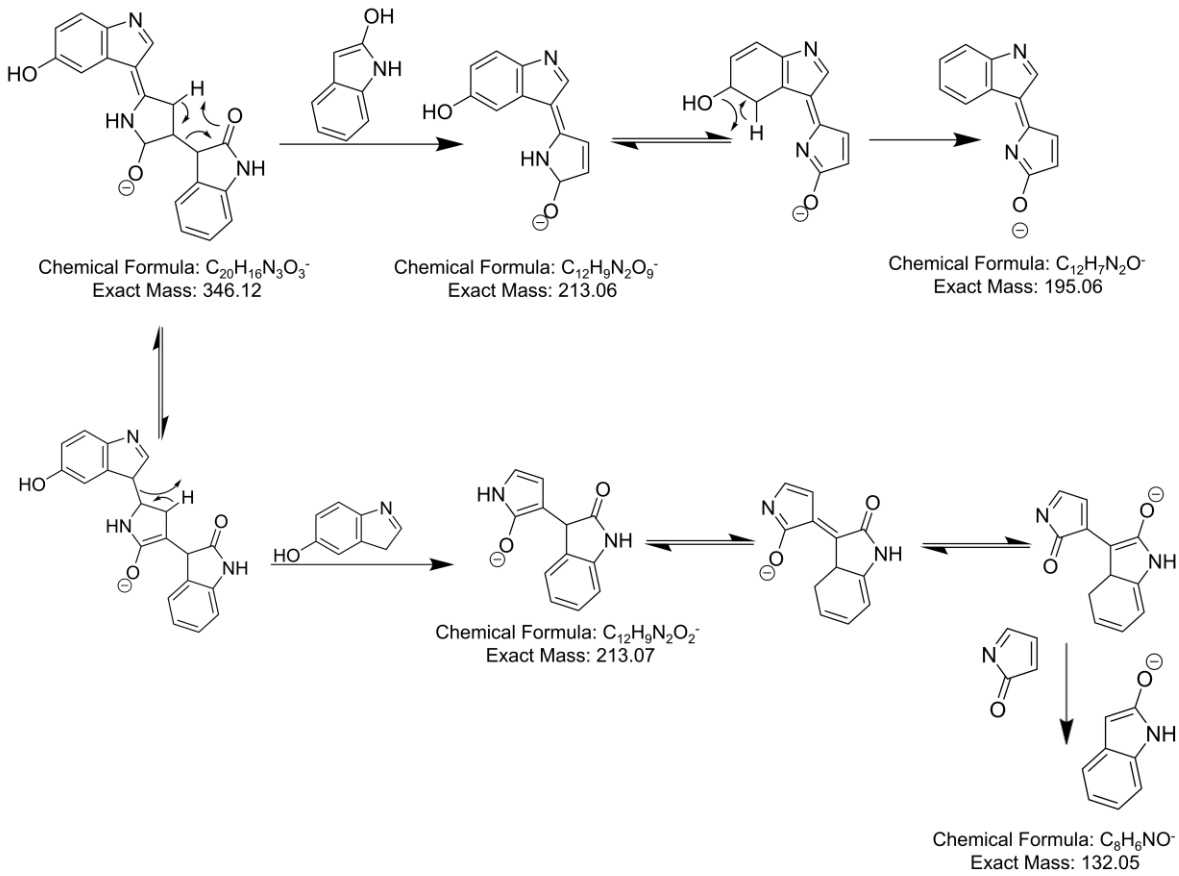

Pathway II: Ion *m*/*z* 346 undergoes a remote hydrogen rearrangement, leading to the ring opening of the pentacyclic amide and a retro-ene reaction, resulting in the elimination of $C_{10}H_9NO$ and the formation of fragment *m*/*z* 187. Additionally, a retro-ene reaction leads to the formation of ion *m*/*z* 158 through the neutral loss of $C_{10}H_8N_2O_2$.

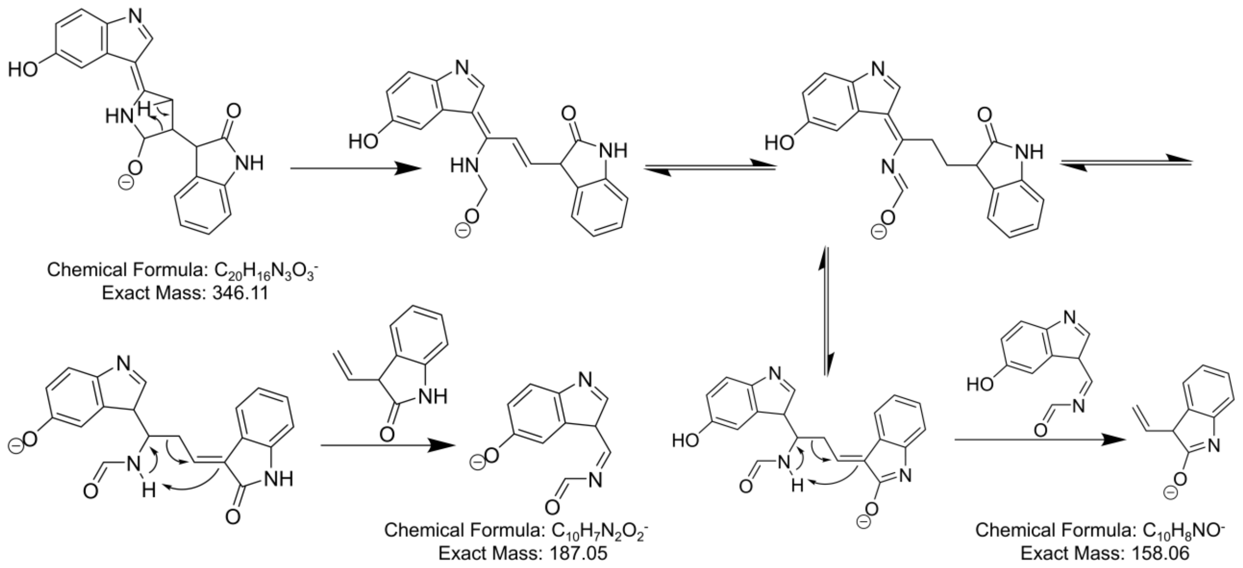

Figure S5. Violacein-Reduced-Reduced fragmentation pathway – M4 – Negative electrospray ionization mode – MS/MS.

Pathway I: Ion *m*/*z* 348 undergoes a neutral loss of water, followed by a retro-heteroene reaction, leading to the elimination of C_8H_7NO and the formation of fragment *m*/*z* 197.

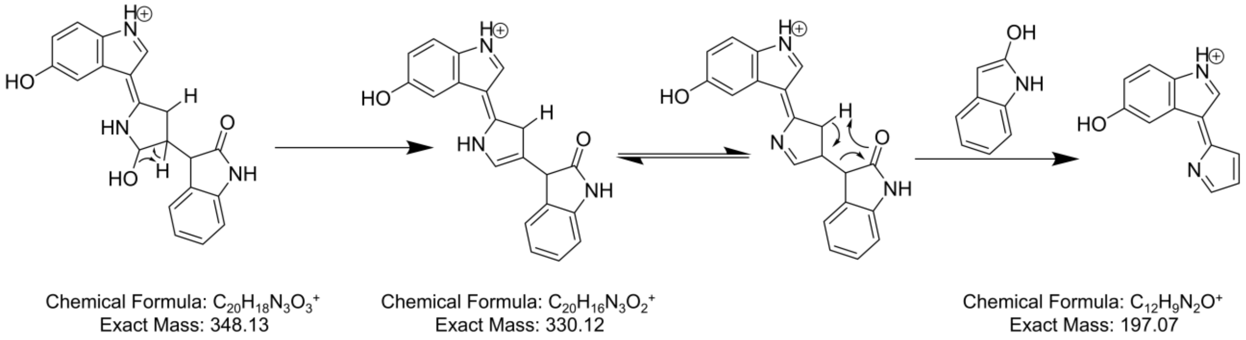

Pathway II: The mechanism in black begins with ion *m*/*z* 348 undergoing a remote hydrogen rearrangement, leading to the formation of ion *m*/*z* 270 with the elimination of benzene. In addition, ion *m*/*z* 348, in blue, undergoes ring opening of the pentacyclic amide, followed by a sigmatropic hydrogen rearrangement and a neutral loss of ammonia, resulting in the formation of fragment *m*/*z* 331.

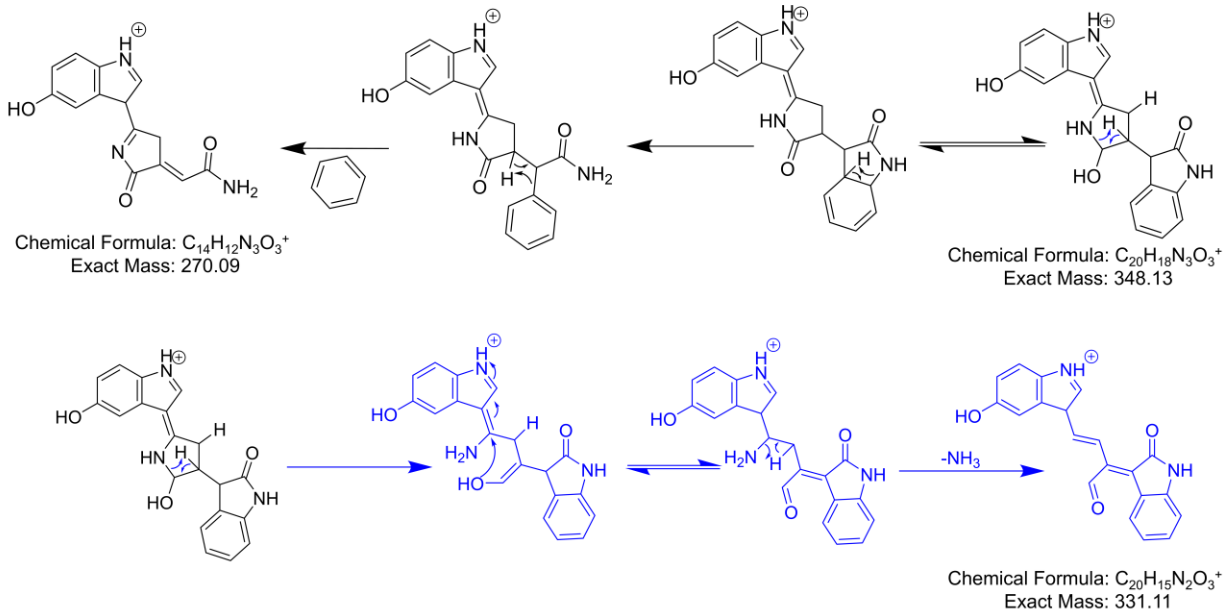

Figure S5 Continued. Violacein-Reduced-Reduced fragmentation pathway – M4– Positive electrospray ionization mode – MS/MS - Pathway I and Pathway II.

Pathway III: Ion *m*/*z* 348 can undergo a neutral loss of C_8H_7NO, leading to the formation of ion *m*/*z* 198, and a neutral loss of CO, resulting in ion *m*/*z* 170.In parallel, ion *m*/*z* 348 can also eliminate NH_3, leading to the formation of ion *m*/*z* 331.

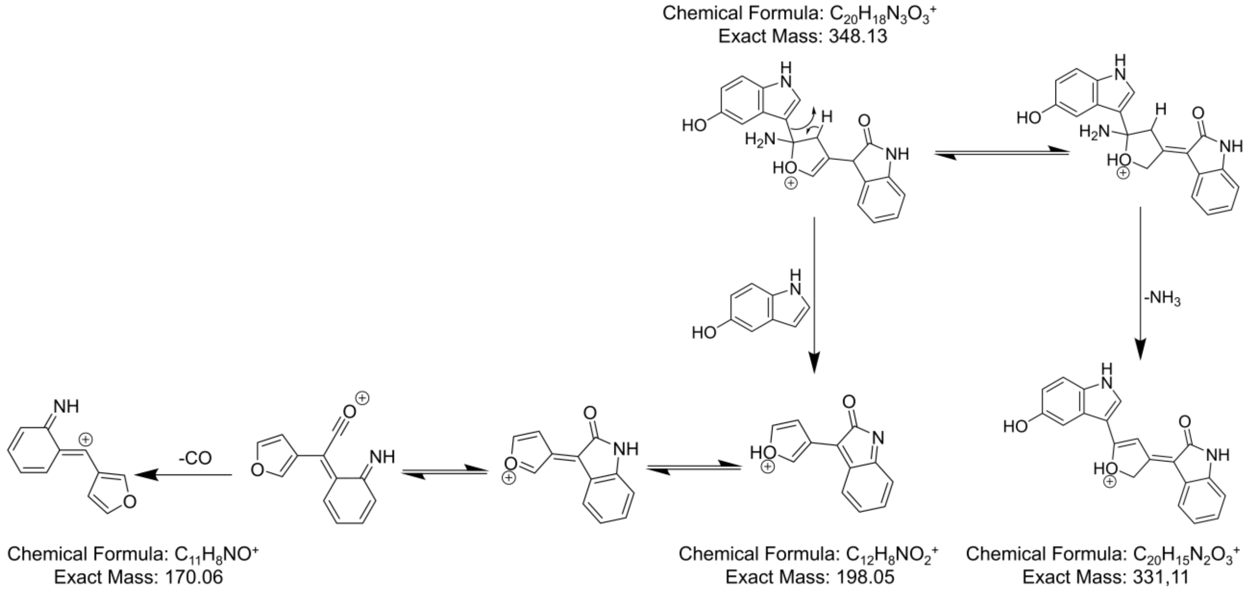

Figure S5 Continued. Violacein-Reduced-Reduced fragmentation pathway – M4 – Positive electrospray ionization mode – MS/MS - Pathway I and Pathway III.
